# The Genetic Architecture of Hearing Impairment in Mice: Evidence for Frequency-Specific Genetic Determinants

**DOI:** 10.1534/g3.115.021592

**Published:** 2015-09-04

**Authors:** Amanda L. Crow, Jeffrey Ohmen, Juemei Wang, Joel Lavinsky, Jaana Hartiala, Qingzhong Li, Xin Li, Pezhman Salehide, Eleazar Eskin, Calvin Pan, Aldons J. Lusis, Hooman Allayee, Rick A. Friedman

**Affiliations:** * Department of Preventive Medicine and Institute for Genetic Medicine, Keck School of Medicine, University of Southern California, Los Angeles, California 90033; † House Ear Institute, Los Angeles, California 90057; ‡ Department of Otolaryngology and Zilkha Neurogenetic Institute, Keck School of Medicine, University of Southern California, Los Angeles, California 90033; § Department of Otolaryngology, Head and Neck Surgery, Eye & ENT Hospital of Fudan University, Shanghai 200031, China; ** Clinical Laboratory Department, First Affiliated Hospital of Nanchang University, Nanchang, Jiangxi Province 330006, China; †† Department of Computer Science and Inter-Departmental Program in Bioinformatics, University of California, Los Angeles, California 90095; ‡‡ Departments of Human Genetics, Medicine, and Microbiology, Immunology, and Molecular Genetics, David Geffen School of Medicine at UCLA, Los Angeles, California 90095

**Keywords:** genome-wide association study (GWAS), Hybrid Mouse Diversity Panel (HMDP), genetics, genomics, ABR, hearing, cochlear function

## Abstract

Genome-wide association studies (GWAS) have been successfully applied in humans for the study of many complex phenotypes. However, identification of the genetic determinants of hearing in adults has been hampered, in part, by the relative inability to control for environmental factors that might affect hearing throughout the lifetime, as well as a large degree of phenotypic heterogeneity. These and other factors have limited the number of large-scale studies performed in humans that have identified candidate genes that contribute to the etiology of this complex trait. To address these limitations, we performed a GWAS analysis using a set of inbred mouse strains from the Hybrid Mouse Diversity Panel. Among 99 strains characterized, we observed approximately two-fold to five-fold variation in hearing at six different frequencies, which are differentiated biologically from each other by the location in the cochlea where each frequency is registered. Among all frequencies tested, we identified a total of nine significant loci, several of which contained promising candidate genes for follow-up study. Taken together, our results indicate the existence of both genes that affect global cochlear function, as well as anatomical- and frequency-specific genes, and further demonstrate the complex nature of mammalian hearing variation.

Genome-wide association studies (GWAS) have been successfully applied to numerous complex traits in humans (Welter *et al.* 2014). These GWAS leverage natural genetic variation in an unbiased approach that is advantageous for studying complex traits. We and others have shown that audiometric threshold elevation secondary to aging in humans is continuously distributed throughout the population (Friedman *et al.* 2009) and that the human cochlea has a perceptual frequency spectrum of 20 Hz to 20 kHz. Heritability studies have shown that the sources of this variance are both genetic and environmental, with approximately half of the variance attributable to hereditary factors (Huang and Tang 2010). As such, a major impediment to progress in the discovery of risk loci for hearing in humans is the lack of control of the many environmental factors that affect hearing during the lifetime, and, to date, only a limited number of large-scale GWAS for hearing phenotypes have been undertaken in humans. We recently reported an association between age-related hearing loss and SNPs within the GRM7 gene and subsequently demonstrated, in a more comprehensive study, the polygeneic nature of this disease (Fransen *et al.* 2015). Despite these successes, association studies in humans (particularly for common forms of hearing loss and baseline hearing ability) are still plagued by small sample sizes and phenotypic heterogeneity.

To harness the same investigative power of the GWAS approach in a more controlled environment that addresses some limitations of human studies, several groups have proposed mouse GWAS (Valdar *et al.* 2006; Bennett *et al.* 2010; Flint and Eskin 2012; Ghazalpour *et al.* 2012; Mott and Flint 2013). For obvious reasons, mouse models have several advantages over human studies. The environment can be more carefully controlled, measurements can be replicated in genetically identical animals, and the proportion of the variability explained by genetic variation is increased. Complex traits in mouse strains have been shown to have higher heritability, and genetic loci often have stronger effects on the trait compared to humans (Lindblad-Toh *et al.* 2000; Wiltshire *et al.* 2003; Yalcin *et al.* 2004). Furthermore, several recently developed strategies for mouse genetic studies, such as use of the Hybrid Mouse Diversity Panel (HMDP), provide much higher resolution for associated loci than traditional approaches with quantitative trait loci (QTL) mapping (Bennett *et al.* 2010; Ghazalpour *et al.* 2012). The genetic and functional similarities of the mouse and human ear coupled with the ability to control environmental exposure make the mouse the ideal model for the study of age-related hearing loss.

Both naturally occurring and experimentally induced mutations in mice have provided much of our current understanding of many diseases of the ear. It has long been our hypothesis that common forms of hearing impairment in both mice and humans is a complex trait resulting from susceptibility loci throughout the genome that contribute to the natural variation in hearing over time. Based on literature suggesting that most common disease phenotypes result from sequence variation, often regulatory, in genes that are different from those underlying Mendelian traits, *e.g.*, nonsyndromic hearing loss, we undertook a study to characterize the genetics of hearing by performing an association analysis that exploits the natural genetic and phenotypic variation of hearing levels in inbred strains of mice.

## Materials and Methods

### Animals

Female 5-wk-old mice of 99 inbred strains (n = 3–8) from the HMDP (Jackson Laboratories) were obtained and used in accordance with the University of Southern California Institutional Animal Care and Use Committee (IACUC) guidelines. Mice were housed in sterilized microisolator cages and received autoclaved food and water *ad libitum*.

### Hearing assessment using auditory brainstem response thresholds (ABR)

Mice were anesthetized with an intraperitoneal injection of a mixture of ketamine (80 mg/kg body weight) and xylazine (16 mg/kg body weight). Mouse body temperature was maintained through the use of a TCAT-2DF temperature controller and the HP-4 M heating plate (Physitemp Instruments Inc., Clifton, NJ). Artificial tear ointment was applied to the eyes during anesthesia. Finally, mice recovered from anesthesia on a heating pad. Stainless-steel electrodes were placed subcutaneously at the vertex of the head and the left mastoid. A ground electrode was placed at the base of the tail. Test sounds were presented using an Intelligent Hearing Systems speaker attached to an 8-inch-long tube that was inserted into the ear canal. Due to time and equipment constraints, only the left ear was assessed.

Auditory signals were presented as tone pips (4, 8, 16, 24, and 32 kHz) in the form of a hamming wave with a 0.3-ms rise and fall time (total time of 1 ms). These signals were presented at a rate of 40 per second. They were then sent to an amplifier and then to the sound transducer from Intelligent Hearing Systems. Physiologic responses were recorded with a 20,000 analog-to-digital rate and sent to an eight-channel 150-gain AC/DC headbox, and then onto a secondary Synamps signal amplifier of 2500 gain before analysis. Filter settings were set at a low pass of 3000 Hz and a high pass of 100 Hz with an artifact rejection of signals with amplitudes exceeding ±50 μV. Three thousand waveforms were averaged at each stimulus intensity. Tone bursts were first presented at a high intensity to elicit a waveform. Next, the intensity was decreased by 20 dB until nearing threshold. Intensity was then decreased in smaller steps of 10, 5, and 2 dB as threshold was approached. Hearing threshold was determined by visual inspection of ABR waveforms and was defined as the intensity at which two peaks could be distinguished. Experiments were duplicated at low intensities when the peaks were not apparent.

### RNA isolation and microarray analysis

We isolated cochlea from 64 HMDP strains (2–4 mice per strain) mice at age 6 wk. RNA was purified after decapitation, the inner ear was microdissected, all soft tissue was removed from the cochlear capsule, and the vestibular labyrinth was removed at the level of the round and oval windows. The microdissected cochleae were frozen in liquid nitrogen, ground to powder under liquid nitrogen, and RNA lysis buffer was added (Ambion). The sample was incubated over night at 4°, centrifuged at 12,000 × *g* for 5 min to pellet insoluble materials, and RNA was isolated following manufacturer’s recommendations. This procedure yielded approximately 300 ng of total RNA per animal. Gene expression data were generated using Illumina’s Mouse whole genome expression BeadChips (MouseRef-8-v1 Expression BeadChip). All amplifications and hybridizations were performed according to Illumina’s protocol by the Southern California Genome Consortium microarray core laboratory at UCLA. In brief, 100 ng of total RNA was reverse-transcribed to cDNA using Ambion cDNA synthesis kit AMIL1791 and then converted to cRNA and labeled with biotin. Then, 800 ng of biotinylated cRNA product was hybridized to prepared whole genome arrays and allowed to incubate overnight (16–20 hr) at 55°. Arrays were washed and then stained with Cy3-labeled streptavadin. Excess stain was removed by washing and the arrays were dried and scanned on an Illumina BeadScan confocal laser scanner.

### Association analyses for hearing phenotypes and tissue eQTLs

For eQTLs in the liver, we used previously generated expression data in the HMDP (http://geneeqtl.genetics.ucla.edu/).

GWAS analyses for transcript abundance in the cochlea and hearing phenotypes in the HMDP strains were performed using genotypes of ∼450,000 SNPs obtained from the Mouse Diversity Array (Yang *et al.* 2009). SNPs were required to have minor allele frequencies >5% and missing genotype frequencies <10%. Applying these filtering criteria resulted in a final set of ∼200,000 SNPs that were used for analysis. Association testing was performed using FaST-LMM (Lippert *et al.* 2011), a linear mixed model method that is fast and able to account for population structure. To improve power, when testing all SNPs on a specific chromosome, the kinship matrix was constructed using the SNPs from all other chromosomes. This procedure includes the SNP being tested for association in the regression equation only once. Genome-wide significance threshold in the HMDP was determined by the family-wise error rate (FWER) as the probability of observing one or more false positives across all SNPs per phenotype. We ran 100 different sets of permutation tests and parametric bootstrapping of size 1000 and observed that the genome-wide significance threshold at a FWER of 0.05 corresponded to *P*=4.1×10^−6^, similar to what has been used in previous studies with the HMDP (Ghazalpour *et al.* 2012). This is approximately an order of magnitude larger than the threshold obtained by Bonferroni correction (4.6×10^−7^), which would be an overly conservative estimate of significance because nearby SNPs among inbred mouse strains are highly correlated with each other.

### Data availability

Strains and genotype data are available from Jackson Labs (http://jax.org).

## Results

### Variation in ABR thresholds in the HMDP

**Figure 1:**
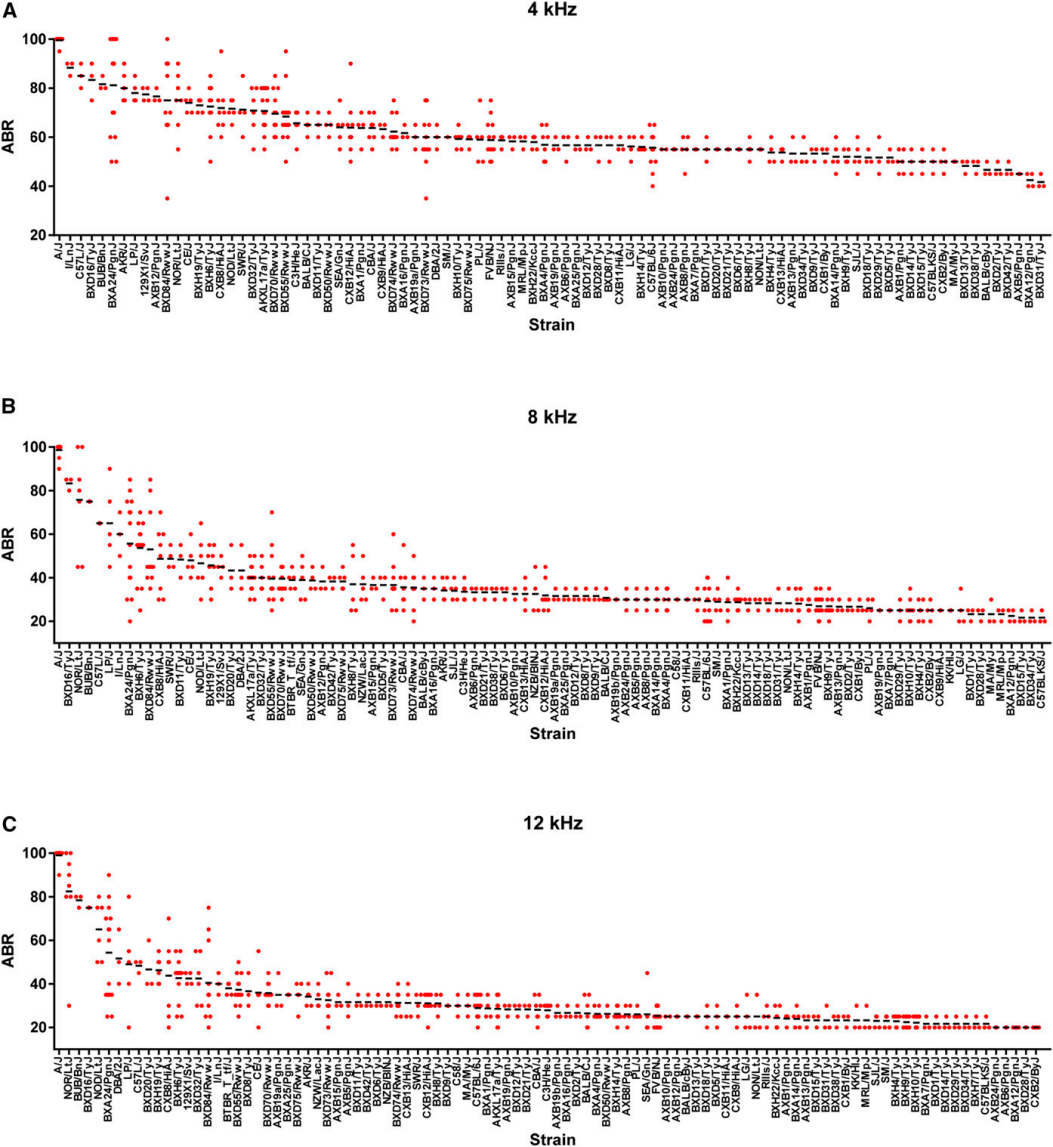

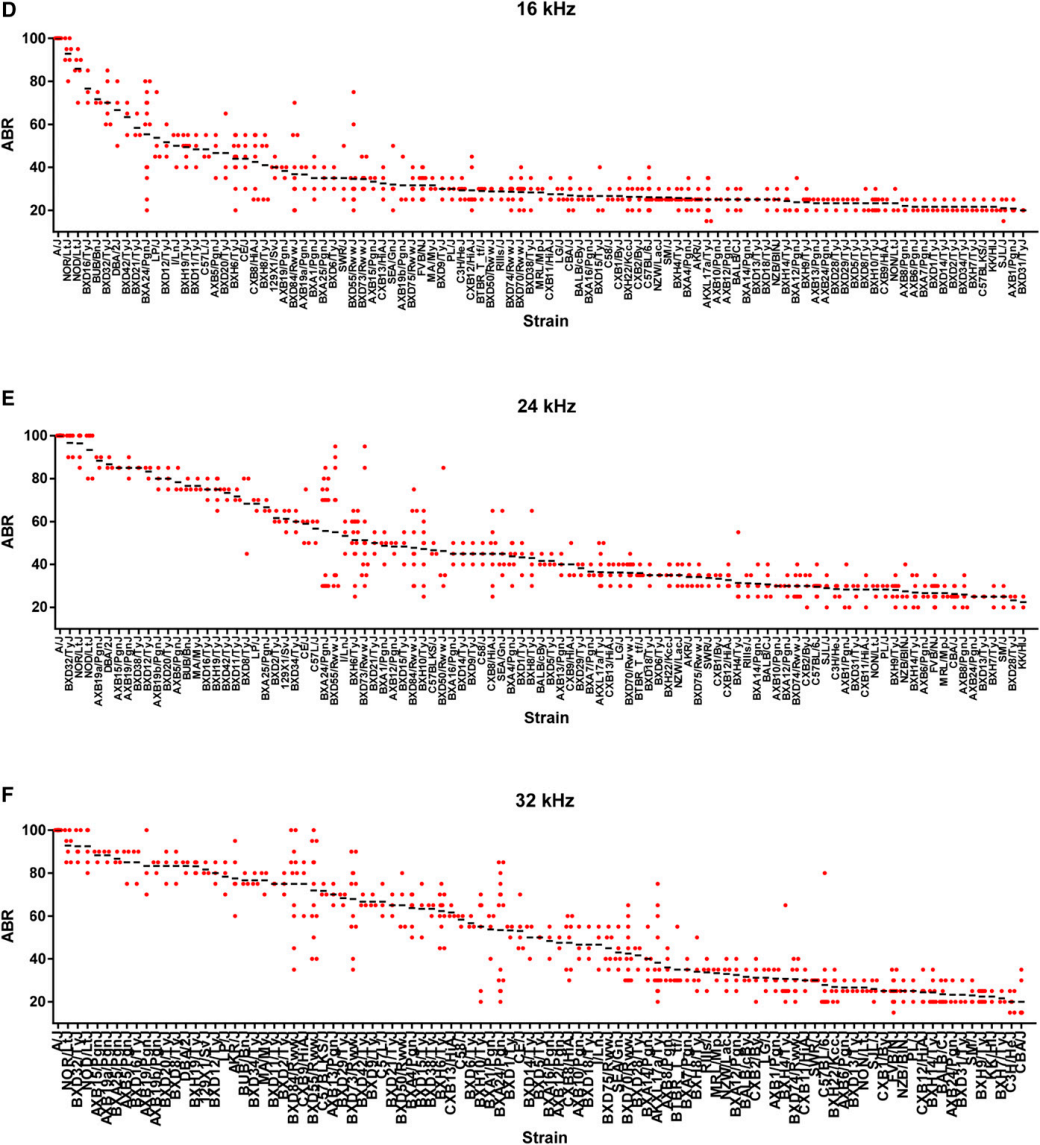
Variation in ABR in the HMDP. The auditory brainstem response (ABR) to tone burst stimuli at six different frequencies exhibits two-fold to five-fold variation among 99 HMDP strains. Each dot represents an individual mouse from the respective strain and the mean values are indicated by the horizontal black bars. Female 5-wk-old mice were exposed to auditory signals at frequencies of 4 kHz, 8 kHz, 12 kHz, 16 kHz, 24 kHz, and 32 kHz. The ability of individual mice to hear these signals was assessed using an ABR test. ABR is represented by the decibel level at which hearing threshold was reached, determined visually by an ABR waveform.

As part of our effort to comprehensively characterize the genetic basis of strain variation in hearing, we recorded ABR threshold data in 5- to 6-wk-old female mice from 29 common inbred (CI) and 70 recombinant inbred (RI) mouse strains from the HMDP. Figure 1, A–F demonstrates the results for ABR thresholds after tone burst stimuli of 4, 8, 12, 16, 24, and 32 kHz in the HMDP. Notably, among the 99 strains characterized, ABR across the frequencies tested varied by two-fold to five-fold, strongly suggesting a complex underlying genetic architecture. Furthermore, ABR thresholds were highly correlated, particularly among those across frequencies at the same range of the spectrum (Supporting Information, Figure S1, Figure S2).

### GWAS for ABR threshold variation at each tested frequency

**Figure 2:**
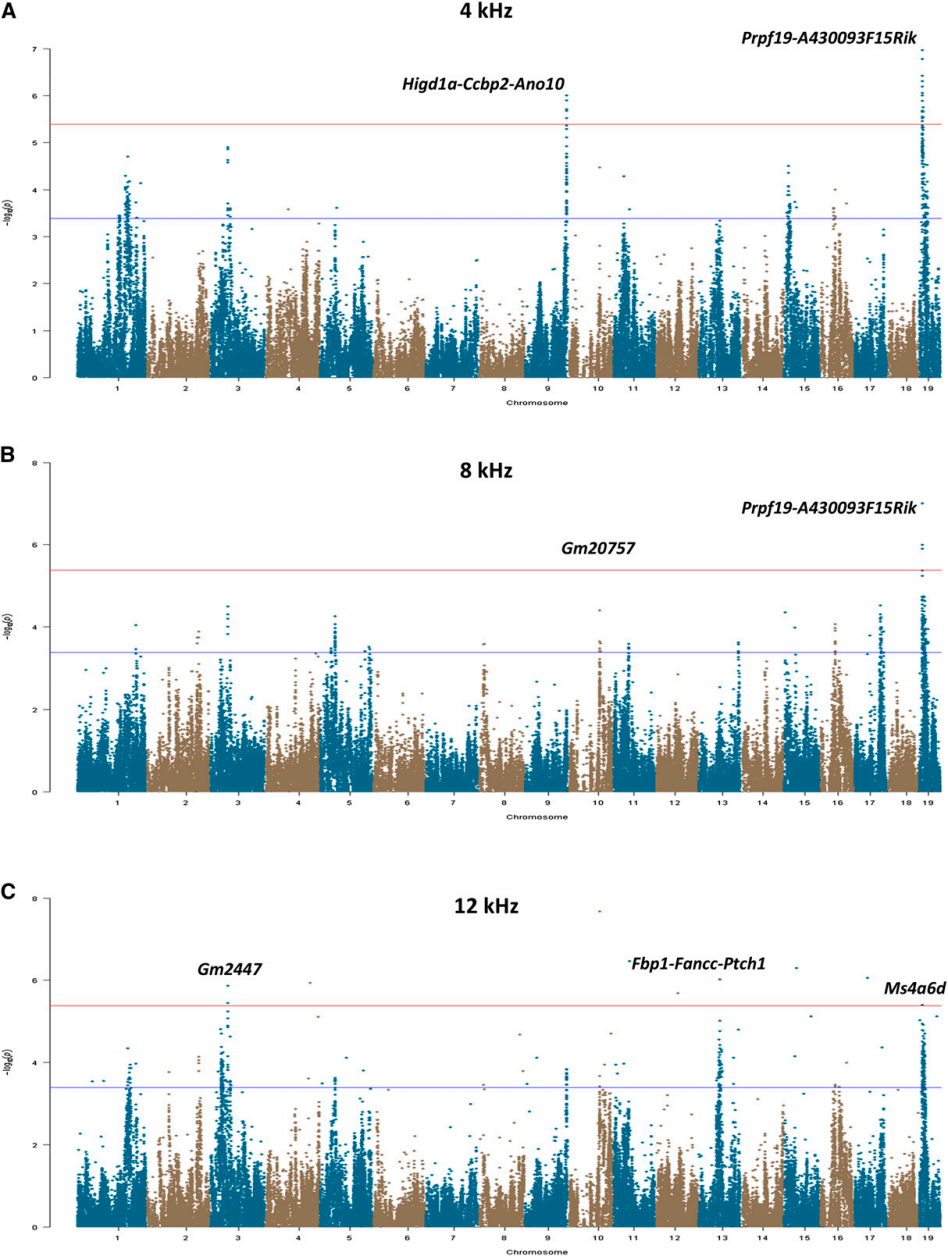

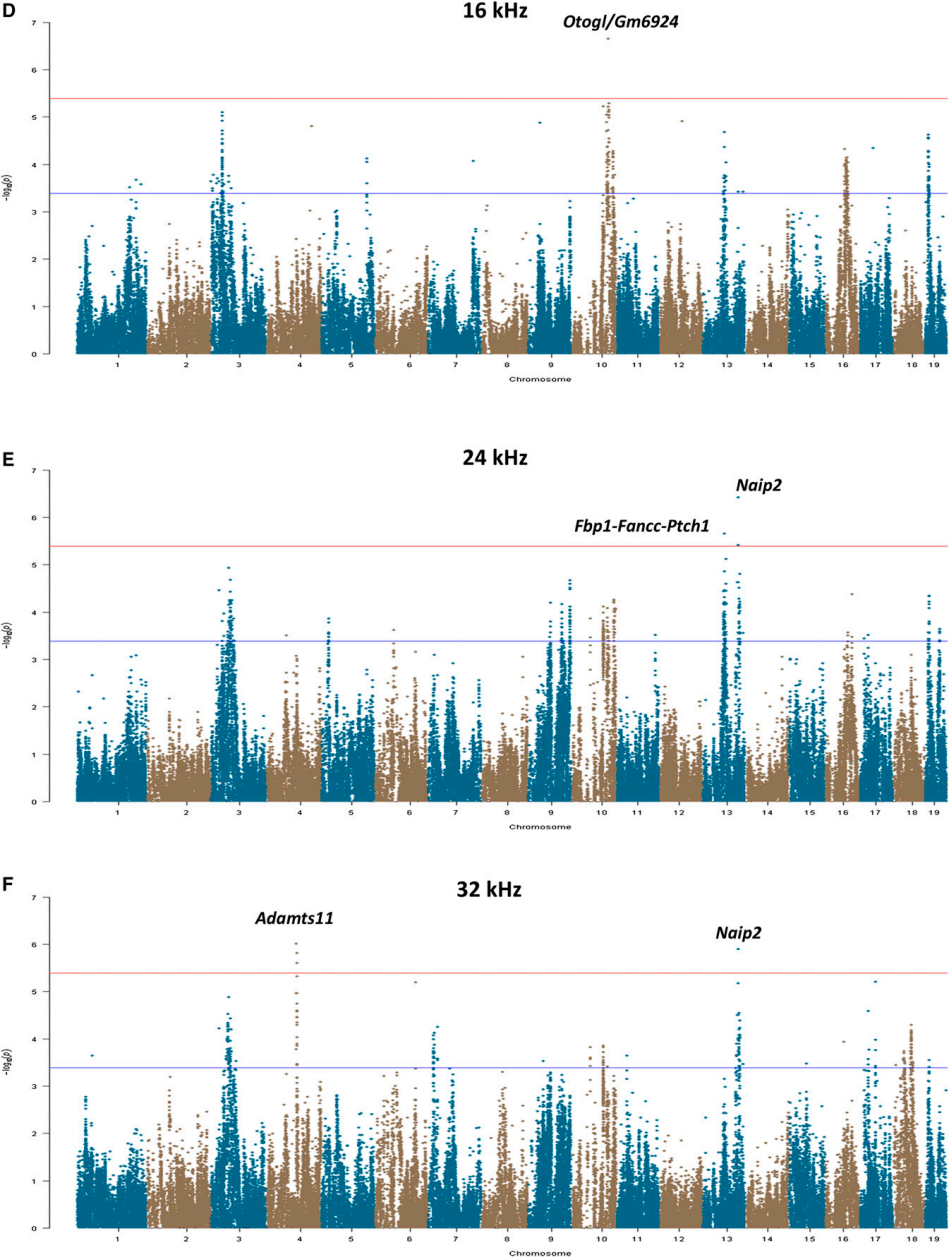
Manhattan plots of GWAS results for ABR at each tone burst frequency. (A) ABR at 4 kHz was significantly associated with a locus on chromosome 9 and a region on chromosome 19. (B) A GWAS for the 8-kHz tone burst revealed two significantly associated loci on chromosomes 10 and 19. (C) Three significantly associated loci on chromosomes 3, 13, and 19 were identified in the GWAS analysis for the 12-kHz tone burst. (D) One significant locus on chromosome 10 was associated with ABR at the 16-kHz tone burst. (E) The GWAS for ABR at 24 kHz identified two different significant loci on chromosome 13. (F) Two significant loci on chromosomes 4 and 13 were associated with ABR at 32 kHz. For each significantly associated locus, the gene(s) nearest to the peak SNP is indicated. The GWAS analyses for each of the six tone burst stimuli included 182,270 SNPs, whose genomic positions are shown along the x-axis with their corresponding -log10 *P* values indicated by the y-axis. The genome-wide thresholds for significant (*P*=4.1×10^−6^) and suggestive (*P*=4.1×10^−4^) evidence of association are indicated by the horizontal red and blue lines, respectively.

Based on the phenotypic data in the HMDP, we next sought to identify the genetic factors affecting hearing by performing a GWAS for ABR thresholds at each tone burst stimulus frequency. These analyses used an efficient mixed model algorithm (EMMA) that takes into account the underlying population structure and genetic relatedness of the HMDP strains (Kang *et al.* 2008) and has been used successfully for association mapping in the HMDP. These GWAS analyses yielded several interesting observations (Figures 2, A–2F.) First, we identified at least one locus for each frequency that achieved genome-wide significance, with a total of nine regions distributed on chromosomes 3, 4, 9, 10, 13, and 19 (Table 1). Second, with the exception of the 24 kHz stimulus, each analysis identified at least one locus that was frequency-specific. Third, a locus on chromosome 3 (rs30259360) that was significantly associated with ABR threshold after a 12-kHz stimulus also demonstrated a suggestive association (*P*∼10^−5^) with nearly all other ABR traits (Table 1). Fourth, loci that were associated with ABR threshold across multiple frequencies tended to cluster at the same end of the spectrum. For example, the chromosome 19 locus (rs30354441) that was associated with ABR threshold after a 4-kHz tone burst also demonstrated association after the 8-kHz stimulus but not the other frequencies. Conversely, a locus on chromosome 13 (rs52344209) was only common to ABR threshold after 24-kHz and 32-kHz tone bursts. Finally, one locus on chromosome 10 (rs29362366), which was associated with ABR threshold only after a 16-kHz stimulus, maps to *Otogl*, a gene that has been implicated in a Mendelian form of human deafness (Yariz *et al.* 2012).

**Table 1:** Loci identified in HMDP for ABR thresholds at various tone burst frequencies

Trait	Chr	Position (bp)	Lead SNP	95% CI (Mb)	Candidate Gene(s)	MAF	*P*
**4 kHz**	3	52509617	rs30259360	51.2, 54.8	*Gm2447*	0.50	1.3×10^−5^
	9	122415312	rs33514183	121.1, 123.7	*Higd1a-Ccbp2-Ano10*	0.39	**9.9×10^−7^**
	19	10834438	rs30354441	9.5, 11.1	*Prfp19-A430093F15Rik*	0.06	**1.1×10^−7^**
**8 kHz**	3	52509617	rs30259360	51.2, 54.8	*Gm2447*	0.50	3.2×10^−5^
	10	91728327	rs38135766	90.4, 93.0	*Gm20757*	0.28	**1.2×10^−6^**
	19	10834438	rs30354441	9.5, 11.1	*Prfp19-A430093F15Rik*	0.06	**9.9×10^−8^**
**12 kHz**	3	52509617	rs30259360	51.2, 54.8	*Gm2447*	0.50	**1.4×10^−6^**
	13	63603934	rs51991909	62.3, 64.9	*Fbp1-Fancc-Ptch1*	0.28	**9.6×10^−7^**
	19	11217919	rs36642183	9.9, 12.5	*Ms4a6d*	0.18	**4.1×10^−6^**
**16 kHz**	10	107329794	rs29362366	9.4, 12.0	*Otogl/Gm6924*	0.28	**2.3×10^−7^**
	13	63391591	rs13481847	62.0, 64.6	*Fbp1-Fancc-Ptch1*	0.33	4.3×10^−5^
**24 kHz**	3	52509617	rs30259360	51.2, 54.8	*Gm2447*	0.50	7.4×10^−5^
	13	63391591	rs13481847	62.0, 64.6	*Fbp1-Fancc-Ptch1*	0.33	**2.2×10^−6^**
	13	101851306	rs52344209	100.5, 103.1	*Naip2*	0.44	**3.8×10^−7^**
	3	52509617	rs30259360	51.2, 54.8	*Gm2447*	0.50	1.6×10^−4^
**32 kHz**	4	85925264	rs28095334	84.6, 87.2	*Adamtsl1*	0.22	**9.7×10^−7^**
	13	101851306	rs52344209	100.5, 103.1	*Naip2*	0.44	**1.3×10^−6^**

*P* values exceeding the genome-wide significance threshold (4.1×10^−6^) are shown in bold. Intervals spanning the 95% confidence interval (CI) are given based on previous simulation studies in the HMDP (Bennett *et al.* 2010). Chr, chromosome; MAF, minor allele frequency; BP, base pair position of lead SNP given according to NCBI build 37 of the reference mouse genome sequence.

### Bioinformatics analyses to prioritize positional candidate genes

We next used bioinformatics approaches to prioritize candidate genes at each locus. First, we used publicly available expression data for several tissues in the HMDP (http://geneeqtl.genetics.ucla.edu/) to determine whether variation at the identified loci had *cis*-acting effects on gene expression. Expression QTL (eQTL) were considered local, or *cis*, if the lead SNP mapped within 2 Mb of the peak GWAS SNP. We identified four genes as having *cis* eQTL in the liver: three (*Higd1a*, *Ccbp2*, and *Ano10*) are located in the chromosome 9 locus identified in the 4-kHz GWAS, and the fourth *cis* eQTL in liver is *Naip2*, which is located within the second chromosome 13 region that was identified in the 24 kHz GWAS (Table 2).

**Table 2:** *cis* eQTL for GWAS Loci at Various Tone Burst Frequencies

Trait	Chr	Lead SNP	BP	Nearest Genes	eQTL Lead SNP	eQTL SNP Position	eQTL *P*	eQTL Tissue
**4 kHz**	9	rs33514183	122415312	*Higd1a*	rs13459114	121825029	8.2×10^−20^	Liver
	9	rs33514183	122415312	*Ccbp2*	rs6299531	122834341	2.0×10^−6^	Liver
	9	rs33514183	122415312	*Ano10*	rs3713370	122186398	1.8×10^−11^	Liver
	19	rs30354441	10834438	*Prpf19*	rs30899404	10148177	2.7×10^−6^	Cochlea
**8 kHz**	19	rs30354441	10834438	*Prpf19*	rs30899404	10148177	2.7×10^−6^	Cochlea
**12 kHz**	19	rs36642183	11217919	*Ms4a6d*	rs30768936	11377515	2.7×10^−8^	Cochlea
**24 kHz**	13	rs13481847	63391591	*Fbp1*	rs13481847	63290283	6.1×10^−21^	Cochlea
	13	rs52344209	101851306	*Naip2*	rs3144793	100956146	2.4×10^−11^	Liver

Genomic regions ±1 Mb around the peak SNPs for each frequency were interrogated for the presence of *cis* eQTLs in multiple tissues using the UCLA Systems Genetics Resource (http://systems.genetics.ucla.edu/) and microarray data from cochlear tissue. Only genes exhibiting *cis* eQTLs in cochlea and liver are listed. Chr, chromosome; MAF, minor allele frequency; BP, base pair position of lead SNP given according to NCBI build 37 of the reference mouse genome sequence.

**Figure 3:**
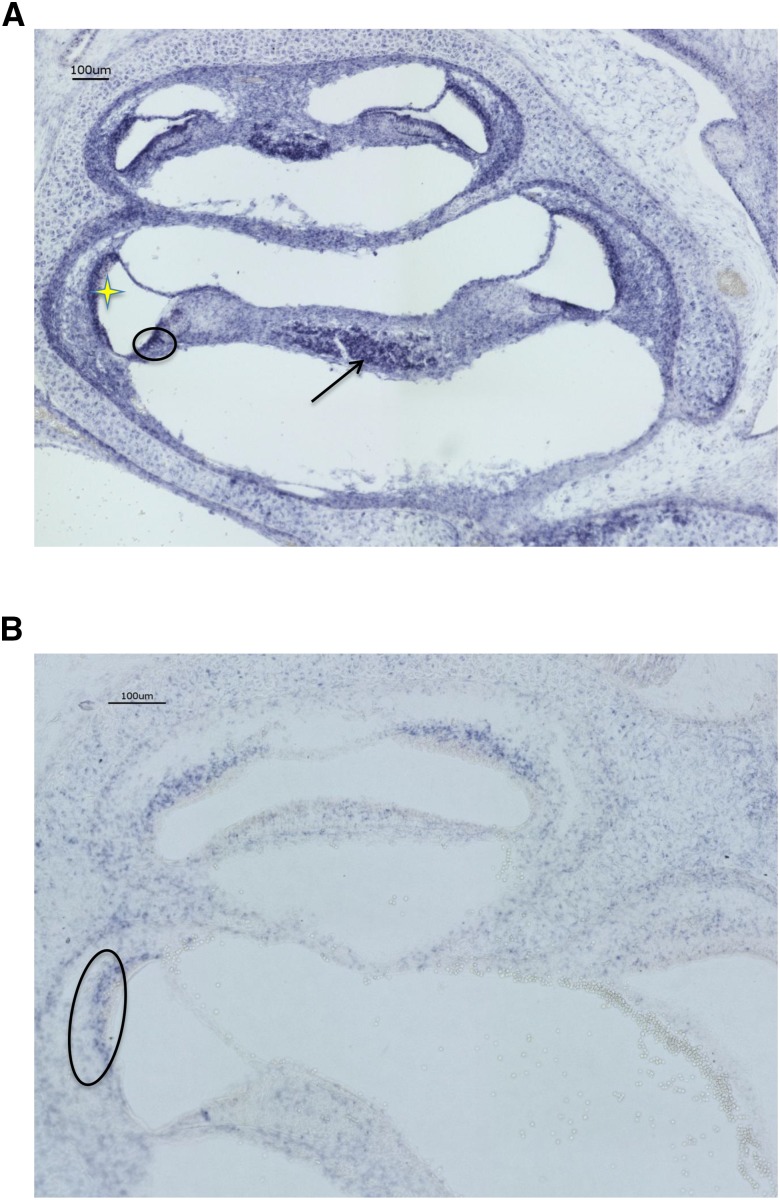
*In situ* images exhibiting cochlear mRNA expression of GWAS candidate genes. (A) Antisense RNA probe for Prpf19 in P1 cochlea demonstrates expression in the cochlear epithelium, represented by the black circle, and spiral ganglion, denoted by the black arrow. Stria vascularis indicated by the yellow star. (B) Antisense RNA probe for Fbp1 in P1 cochlea demonstrates expression in the basal layer of the stria vascularis, represented by the black oval.

Because we are interested in hearing, we wanted to interrogate if any of our genes exhibit *cis* eQTL in a more relevant tissue, namely, the cochlea. Therefore, we performed association analysis on microarray-generated expression data in the cochlea from a subset of 64 HMDP strains. Notably, several of our GWAS loci exhibited *cis* eQTL in cochlear tissue (Table 2). For example, *Prpf19*, which maps approximately 700 kb upstream of our peak GWAS SNP for ABR thresholds after 4 kHz and 8 kHz stimuli, yielded a significant eQTL in the cochlea (rs30899404; *P*=2.7×10^−6^) but not in other tissues. Additionally, an eQTL for *Ms4a6d* (rs30768936; *P*=2.7×10^−8^) was located ∼160 kb downstream of our peak GWAS SNP for the 12-kHz burst in a different region on chromosome 19 (Table 2). Finally, *Fbp1* had a highly significant *cis* eQTL (rs13481847; *P*=6.1×10^−21^) in the cochlea that maps ∼100 kb upstream of our peak GWAS SNP on chromosome 13 for ABR threshold after a 24-kHz tone burst. We also examined *in situ* imaging to visualize where in the P1 mouse cochlea *Prpf19* and *Fbp1* are expressed (Figure 3), as expression in different regions of the organ can indicate different biological functions with respect to hearing. The sensory epithelium and spiral ganglion demonstrate *Prpf19* mRNA expression (Figure 3A); further, *Fbp1* mRNA expression was detected in the basal layer of the striavascularis in P1 mouse cochleae (Figure 3B).

In addition to eQTL analysis, we also used the Ensembl genome browser to determine whether any of the positional candidate genes harbored amino acid substitutions among the inbred mouse strains that were sequenced. Of the candidate genes examined, *Otogl* had an Asn2170Ser polymorphism that was predicted to have functionally deleterious consequences by both SIFT (score = 0.0) and PolyPhen2 (score = 1.0). *Otogl* is 87% identical to the human protein at the amino acid level and Asn2170Ser is located in a region of high homology that is evolutionarily conserved across seven mammalian species (data not shown). Further analysis revealed that *Otogl* harbored four additional amino acid substitutions (Ser177Leu, Ala833Thr, Met1107Ile, Ser1258Pro) among the available inbred strains, but these were similar in their physiochemical properties, computationally predicted by SIFT to be tolerated, or were located in regions of the protein that were not as evolutionarily conserved as the region containing Asn2170Ser (data not shown).

**Figure 4:**
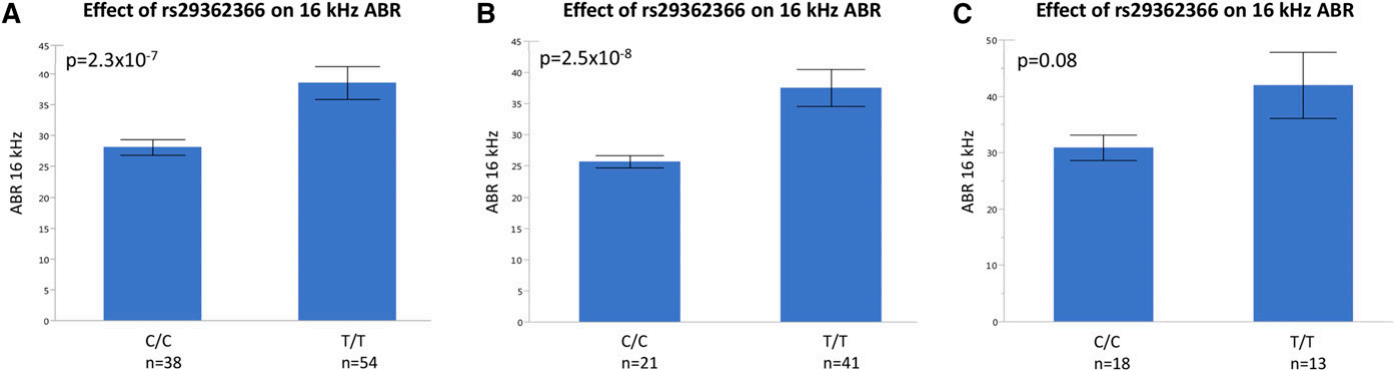
ABR at 16 kHz varies by genotype at rs29362366. (A–C) The effect of *Otogl* peak SNP rs29362366 on 16 kHz ABR. (A) Genotype effect when all strains are included in analysis. (B) Genotype effect when DBA/2J and BXD RI strains derived from C57BL/6J and DBA/2J matings are excluded from analysis. (C) Genotype effect when only C57BL/6J, DBA/2J, and BXD RI strains are included in analysis. Haplotype containing allele C is present in C57BL/6J and haplotype containing allele T is present in DBA/2J.

Based on these data, we reasoned that one or more of these variants may be driving the association signal for ABR at 16 kHz at the *Otogl* locus. Because none of the five amino acid substitutions were included in the panel of SNPs used in our GWAS analyses, we used the publicly available sequence data for ∼25 inbred strains to determine the extent of linkage disequilibrium (LD) between the variants and the peak *Otogl* SNP (rs29362366). This analysis revealed that the Ser177Leu and Ala833Thr variants were in perfect LD with rs29362366, whereas Met1107Ile and Ser1258Pro were much more weakly linked. The Asn2170Ser polymorphism was also only present in strain DBA/2J. Thus, the association at *Otogl* could be due the Ser177Leu and Ala833Thr variants, even though they were not predicted to have functional effects, or other unknown polymorphisms that are present on the haplotype containing Ser177Leu, Ala833Thr, and rs29362366. Alternatively, the association signal could be due to the segregation of Asn2170Ser among DBA and the BXD RI strains. To distinguish between these possibilities, we performed an association analysis with rs29362366 with and without DBA/2J and the BXD strains. Notably, exclusion of these strains increased the significant association of rs29362366 with ABR at 16 kHz by one order of magnitude (*P*=2.5×10^−8^) compared to the analysis that included all HMDP strains (*P*=2.3×10^−7^) (Figure 4). Conversely, rs29362366 was not associated with ABR at 16 kHz in an analysis that included only C57BL/6J, DBA/2J, and the BXD strains (Figure 4). Taken together, these results suggest that the association signal at the *Otogl* locus is due to the haplotype harboring Ser177Leu, Ala833Thr, and rs29362366.

## Discussion

We have used a GWAS approach with correction for population structure to map several loci for hearing traits in inbred strains of mice. Our results identify a number of novel loci and demonstrate two critical additions to the literature. First, with the exception of the *Otogl* locus, which was identified in humans, none of the loci identified in the GWAS with the HMDP overlap with Mendelian loci identified for hearing loss in mice. This observation supports the notion that variation in hearing among inbred mouse strains has a complex genetic architecture. Second, just as the cochlea has a frequency-specific functional place map, we demonstrate that the genetics of hearing sensitivity is frequency-specific as well.

We recently published the first meta-analysis for age-related hearing loss in mice using data from several data sets, including a subset of the HMDP strains (Ohmen *et al.* 2014). The meta-analysis identified five significantly associated loci, including Fscn2, the causative gene at the Ahl8 locus identified in a cross between the DBA/2J and C57BL/6J strains. However, there was no overlap between the loci detected in the meta-analysis and those in the present study. For example, the meta-analysis contained only 35 of the 99 HMDP strains and predominantly powered by the inclusion of N2 mice from a DBA/2J × C57BL/6J backcross generated by Johnson *et al.* (2008). By contrast, the present study sought to identify the genetic determinants for common forms of hearing with the entire HMDP and would thus be more likely to detect allelic variation that is present across all 99 strains. Finally, our current study assessed hearing at 4- and 24-kHz tone burst stimuli, in addition to those at 8, 16, and 32 kHz that were used in our prior study.

### There exists frequency-specific genetic variation in hearing thresholds in inbred strains of mice

One of the most important findings from our analysis is the apparent existence of strong frequency-specific genetic variation in hearing ability across inbred mouse strains. Ruben demonstrated through an autoradiographic study of the mouse cochlea that terminal mitoses in the developing cochlear epithelium were first detected in the cochlear apex (Ruben 1967). From this study it was concluded that the differentiation of the cochlear organ of Corti occurred from the base to the apex. The mammalian cochlea has numerous specializations throughout its length that contribute to the tonotopic representation of perceived sound with the basal portion responding to higher frequencies than the apex (Davis 2003; Mann and Kelley 2011). Critical to the frequency tuning of the cochlea are the morphological and mechanotransductional properties of the hair cells, the basilar membrane, and the spiral ganglion neurons along the length of the cochlear spiral. The molecular bases for these tonotopical specifications along the longitudinal axis of the cochlea remain largely unknown.

The initial observations made by Zheng *et al.* (1999) that different strains exhibit different threshold sensitivities to particular stimulus frequencies were poignant and were a preliminary insight into the strain variation and genetic variation of sensitivities along the length of the cochlear duct. Son *et al.* (2012), using microarray technology, also demonstrated gradients of gene expression in the adult and developing mouse cochlea, a subset of which (approximately 5% of those genes assayed) exhibited clear spatial and temporal variations in expression along the cochlear duct. Utilizing an association-based approach, we build on these prior studies to elucidate the genetic architecture of hearing sensitivities along the length of the cochlear duct in inbred strains of mice. For example, the finding of at least one specific genome-wide significant locus for each frequency in our comprehensive analysis supports this notion. This was true for each tone-burst stimulus tested except 24 kHz. Additional support for frequency-specific, or cochlear regionally specific, genetic determinants comes from the identification of a locus for the lower-frequency apical cochlear spectrum on chromosome 19 (4 kHz and 8 kHz) and of a region on chromosome 13 for the higher frequency basilar cochlear spectrum (24 kHz and 32 kHz). Although a portion of this frequency-specific genetic association can be explained by issues related to study power, we also detected a genome-wide significant locus on chromosome 3 for the 12-kHz tone burst that demonstrated a suggestive association with nearly all other ABR stimulus traits (*P*∼10^−5^).

### Relationship to alternate forms of hearing loss

To date, approximately 18 loci for AHL and noise-induced hearing loss (NIHL) have been described in mice using traditional QTL mapping strategies (http://hearingimpairment.jax.org/table2.html). However, none of the loci we identified overlap with these previously identified regions. There may be several reasons for this observation. First, we tested ABR in mice only at age 5 wk, and many strains used in the previously reported AHL mapping studies do not develop hearing loss until a later age; thus, we would not detect impaired hearing in these strains at 5 wk. Second, prior QTL studies were performed using F2 crosses of two strains of mice, resulting in approximately 50% frequency of each allele in the population of mice for mapping. With the HMDP, however, the effect allele may not be frequent enough among the strains used to provide sufficient power with the linear mixed model mapping algorithm. Despite the limited overlap between previously reported mouse hearing QTL and the association signals in this study, our results may still have clinical relevance and do suggest a connection with hearing phenotypes in humans. For example, bioinformatics and stratified genetic analyses at the chromosome 10 locus implicated *Otogl* as a strong positional candidate for ABR threshold after a 16-kHz stimulus in the HMDP. Interestingly, mutations of the human ortholog, *OTOGL*, have been identified as the cause of Mendelian forms of moderate high-frequency nonsyndromic hearing loss in humans (Yariz *et al.* 20120; Bonnet *et al.* 2013).

### Hearing impairment in mice: natural variation

Our GWAS for hearing phenotypes in mice revealed nine statistically significant candidate loci; of these, bioinformatic analysis suggests prioritizing at least four positional candidate genes for functional validation. As described above, *Otogl* is a strong candidate for the 16-kHz locus and, based on cochlear eQTL analyses, *Prpf19*, *Ms4a6d*, and *Fbp1* represent strong candidates within their respective intervals. *Prpf19* is a pre-mRNA processing factor that has been implicated in mammalian DNA repair (Mahajan and Mitchell 2003) but has no known function in the auditory system. Consistent with the eQTL results, RNAseq data demonstrate a two-fold greater expression level in cochlear hair cells compared to nonsensory cells (N. Segil, personal communication) and our *in situ* data indicate expression in the sensory epithelium and spiral ganglion. Array data similarly confirms two-fold greater expression of *Prpf19* mRNA in the spiral ganglion neurons of mice in comparison to the vestibular ganglion beginning at E16 and extending into adulthood (http://goodrichlabmicroarrays.hms.harvard.edu).

*Ms4a6d* is a member of a multigene four-transmembrane family related to CD20, a hematopoietic cell–specific protein and a high-affinity IgE receptor beta chain (Ishibashi *et al.* 2001) with no known function in the mammalian auditory system. *Ms4a6d* demonstrates nearly four-fold greater levels of expression in nonsensory cells of the inner ear and similar expression levels in the spiral and vestibular ganglia that peak more than 2 wk postnatal, near the time of onset of hearing in mice (N. Segil, personal communication).

*Fbp1* has recently been demonstrated to localize within the cell nucleus in a cell cycle–dependent manner and may be involved in RNA processing, nucleosome assembly, and cell cycle regulation. Like the other putative positional genes described above, it has no known role in hearing in mammals (Mamczur *et al.* 2012). RNAseq data also revealed a two-fold greater expression of this gene in nonsensory cells of the inner ear and variable levels of expression within the spiral and vestibular ganglia of mice during prenatal and postnatal periods); additionally, our *in situ* data place *Fbp1* mRNA in the striavascularis, which is critical to the maintenance of the endocochlear potential. Although a specific function has not been ascribed to the basal cell layer, there are widespread gap junctions between the basal cells and the basal cells and intermediate cells. This suggests that these cells make up a functional syncytium similar to that seen in the myocardium, supporting the notion that this gene may play a role in hearing in mice. Taken together, these results demonstrate that *Prpf19*, *Ms4a6d*, and *Fbp1* are expressed in various cochlear cell types and that the loci harboring these genes contain functional genetic variation with respect to their expression. Additional functional studies will be required to determine whether these genes play a role in mammalian hearing.

In conclusion, we have performed a comprehensive analysis in mice to elucidate the genetic architecture of hearing in response to tone burst stimuli. We identified multiple novel loci that are, for the most part, frequency-specific and illustrate the complex nature of mammalian hearing. By combining systems genetics with bioinformatics, we have also identified plausible positional candidate genes at several loci that can be pursued for functional validation. Importantly, the overlap between the effect of the *Otogl* locus on hearing loss in mice and nonsyndromic hearing loss in humans reinforces the concept that the underlying biological pathways are likely to be conserved among mammals. Furthermore, the results of such genetic studies in mice can potentially be leveraged toward development of novel prevention and treatment strategies.

## 

## Supplementary Material

Supporting Information
