# Supplementary material for: The Genetic Architecture of Hearing Impairment in Mice: Evidence for Frequency-Specific Genetic Determinants
Source: G3 (Bethesda). 2015 Sep 4;5(11):2329–39. doi: 10.1534/g3.115.021592 (PMC4632053; doi:10.1534/g3.115.021592)
Supplement: Supporting Information [file supp_5_11_2329__index.html]

The Genetic Architecture of Hearing Impairment in Mice: Evidence for Frequency-Specific Genetic Determinants — Supporting Information 

# The Genetic Architecture of Hearing Impairment in Mice: Evidence for Frequency-Specific Genetic Determinants

## Supporting Information for Crow *et al.*, 2015

**Files in this Data Supplement:**

- Supporting Information - Figures S1-S2 (PDF, 1 MB)
- Figure S1 - Correlation of ABR between frequencies in the HMDP. (PDF, 553 KB)
- Figure S2 - Regional plots of significant GWAS regions. (PDF, 986 KB)
