# Supplementary material for: The Genetic Architecture of Hearing Impairment in Mice: Evidence for Frequency-Specific Genetic Determinants
Source: G3 (Bethesda). 2015 Sep 4;5(11):2329–39. doi: 10.1534/g3.115.021592 (PMC4632053; doi:10.1534/g3.115.021592)
Supplement: Supporting Information [file supp_g3.115.021592_021592SI.pdf]

G3 · Genes | Genomes | Genetics

The genetic architecture of hearing impairment in mice: evidence for frequency specific genetic determinants.

Amanda L. Crow[1], Jeffrey Ohmen[2], Juemei Wang[3], Joel Lavinsky[3], Jaana Hartiala[1], Qingzhong Li[4], Xin Li[5], Pezhman Salehide [3], Eleazar Eskin[6], Calvin Pan[7], Aldons J. Lusis[7], Hooman Allayee[1], Rick A. Friedman[3]

[1]Department of Preventive Medicine and Institute for Genetic Medicine, Keck School of Medicine, University of Southern California, Los Angeles, CA 90033

[2]House Ear Institute, Los Angeles, CA 90057

[3]Department of Otolaryngology and Zilkha Neurogenetic Institute, Keck School of Medicine, University of Southern California, Los Angeles, CA, 90033

[4]Department of Otolaryngology - Head and Neck Surgery, Eye & ENT Hospital of Fudan University, Shanghai 200031, China

[5]Clinical Laboratory Department, First Affiliated Hospital of Nanchang University, Nanchang, Jiangxi Province 330006, China

[6]Department of Computer Science and Inter-Departmental Program in Bioinformatics, University of California, Los Angeles, Los Angeles, CA 90095

[7]Departments of Human Genetics, Medicine, and Microbiology, Immunology, and Molecular Genetics, David Geffen School of Medicine at UCLA, Los Angeles, CA 90095

Corresponding Author:
Rick A. Friedman
USC Keck School of Medicine
Zilkha Neurogenetic Institute
1501 San Pablo Street (ZNI 231)
Los Angeles, CA 90033
Tel: (323) 442-4843
Fax: (323) 442-2059
Email: rick.friedman@med.usc.edu

DOI: 10.1534/g3.115.021592

Figure S1

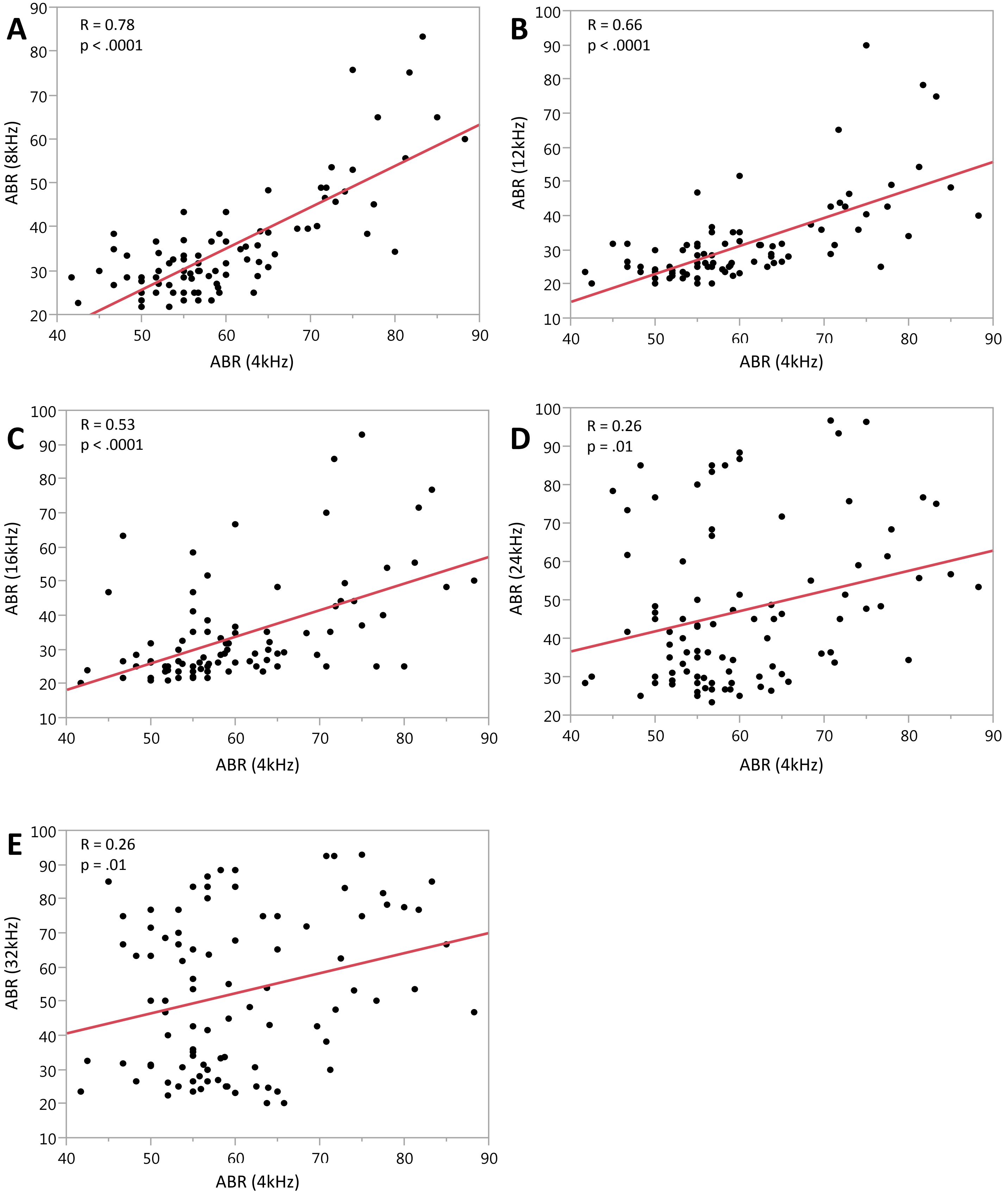
A
R = 0.78
p < .0001
ABR (8kHz)
ABR (4kHz)
B
R = 0.66
p < .0001
ABR (12kHz)
ABR (4kHz)
C
R = 0.53
p < .0001
ABR (16kHz)
ABR (4kHz)
D
R = 0.26
p = .01
ABR (24kHz)
ABR (4kHz)
E
R = 0.26
p = .01
ABR (32kHz)
ABR (4kHz)

Figure S1

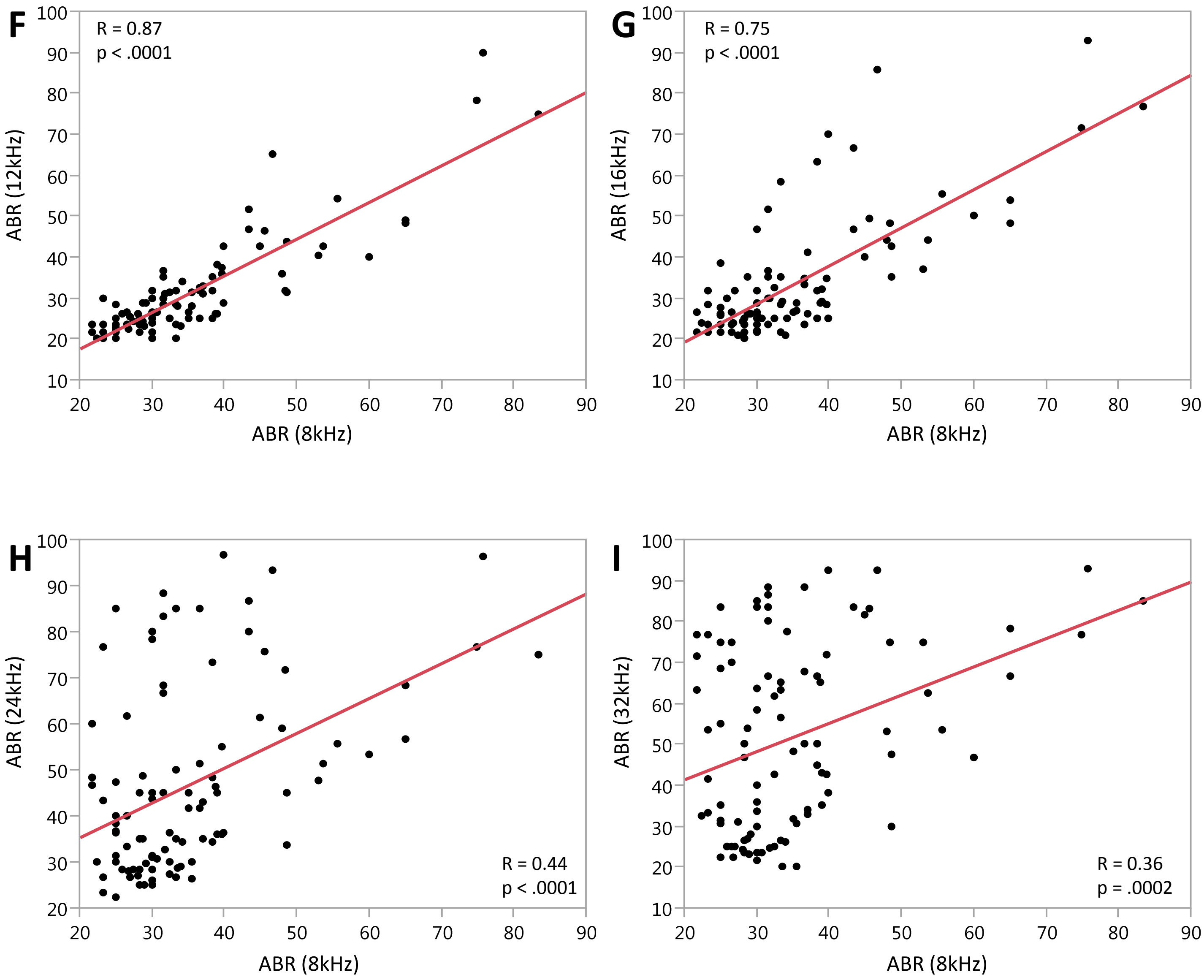
F
R = 0.87
p < .0001
ABR (12kHz)
ABR (8kHz)
G
R = 0.75
p < .0001
ABR (16kHz)
ABR (8kHz)
H
R = 0.44
p < .0001
ABR (24kHz)
ABR (8kHz)
I
R = 0.36
p = .0002
ABR (32kHz)
ABR (8kHz)

Figure S1

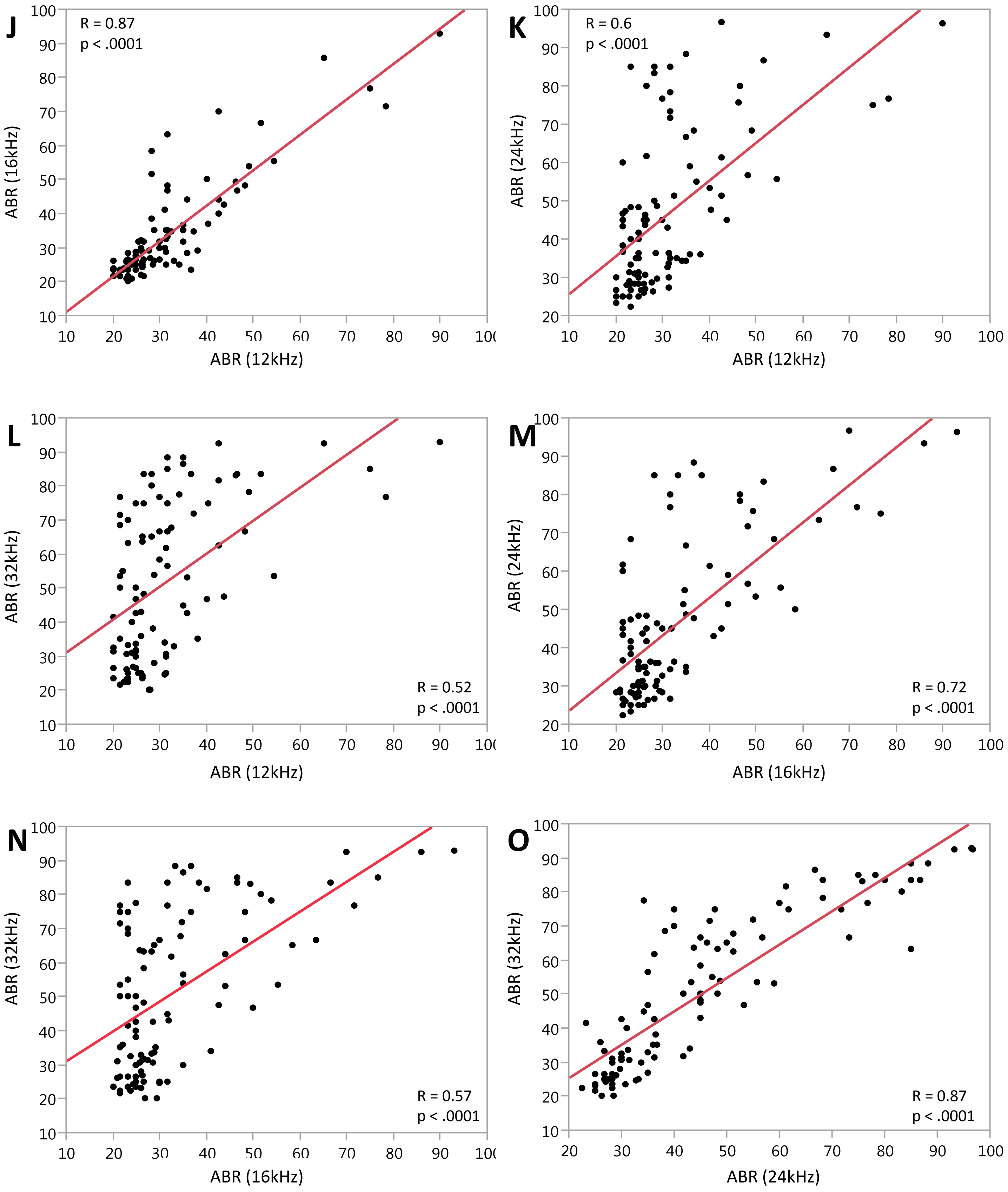
J
R = 0.87
p < .0001
ABR (16kHz)
ABR (12kHz)
K
R = 0.6
p < .0001
ABR (24kHz)
ABR (12kHz)
L
R = 0.52
p < .0001
ABR (32kHz)
ABR (12kHz)
M
R = 0.72
p < .0001
ABR (24kHz)
ABR (16kHz)
N
R = 0.57
p < .0001
ABR (32kHz)
ABR (16kHz)
O
R = 0.87
p < .0001
ABR (32kHz)
ABR (24kHz)

Figure S1. Correlation of ABR between frequencies in the HMDP. Panels A-O demonstrate stronger correlations between mean strain ABR at similar frequencies in the hearing spectrum. Lower (4 kHz-8 kHz), mid-range (8 kHz-12 kHz-16 kHz), and higher (24 kHz-32 kHz) frequencies correlate with each other at R >= 0.75, while comparisons between frequencies outside of their range show much weaker relationships.

Figure S2

4 kHz – Chromosome 9

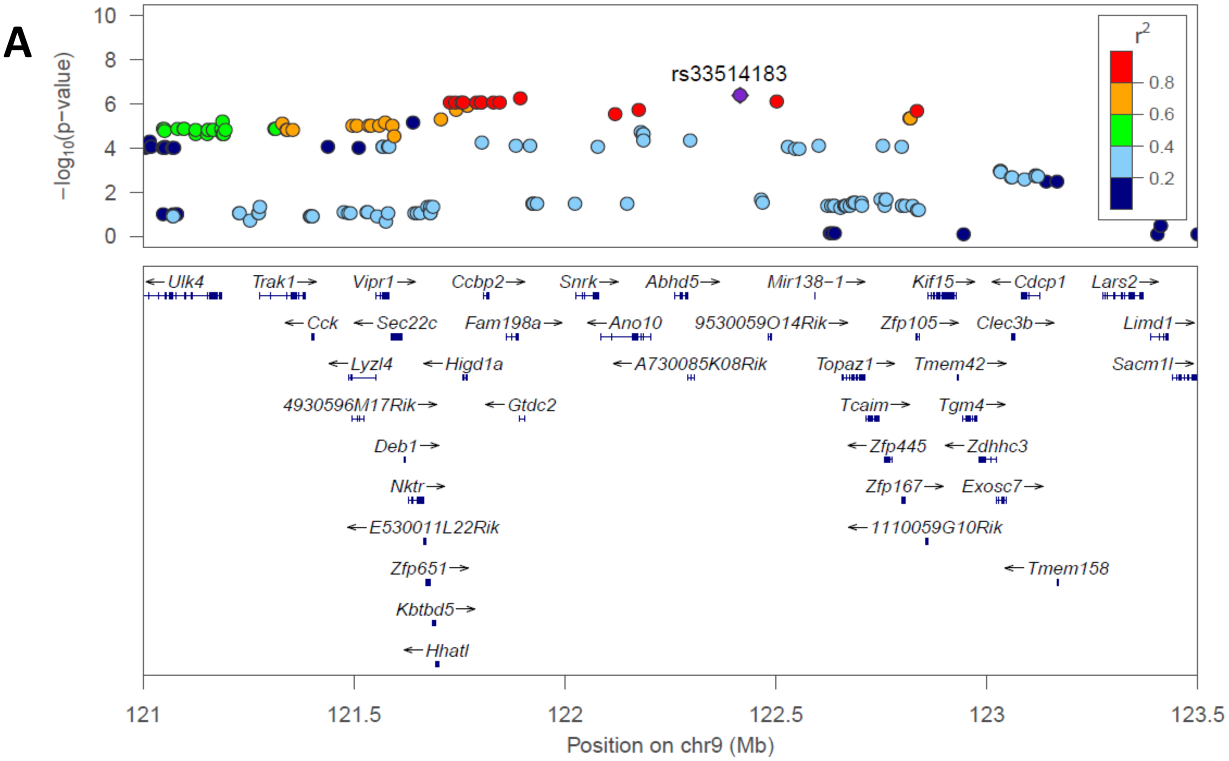

4 kHz – Chromosome 19

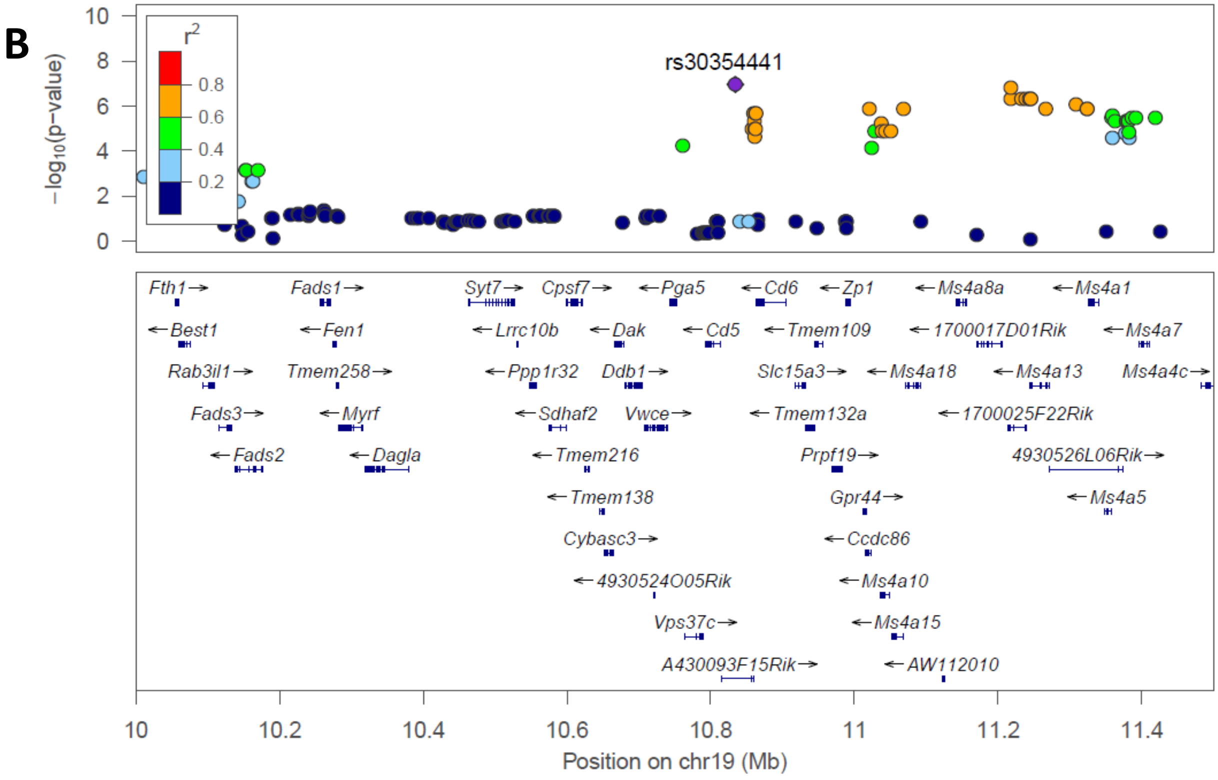

Figure S2

8 kHz – Chromosome 10

C

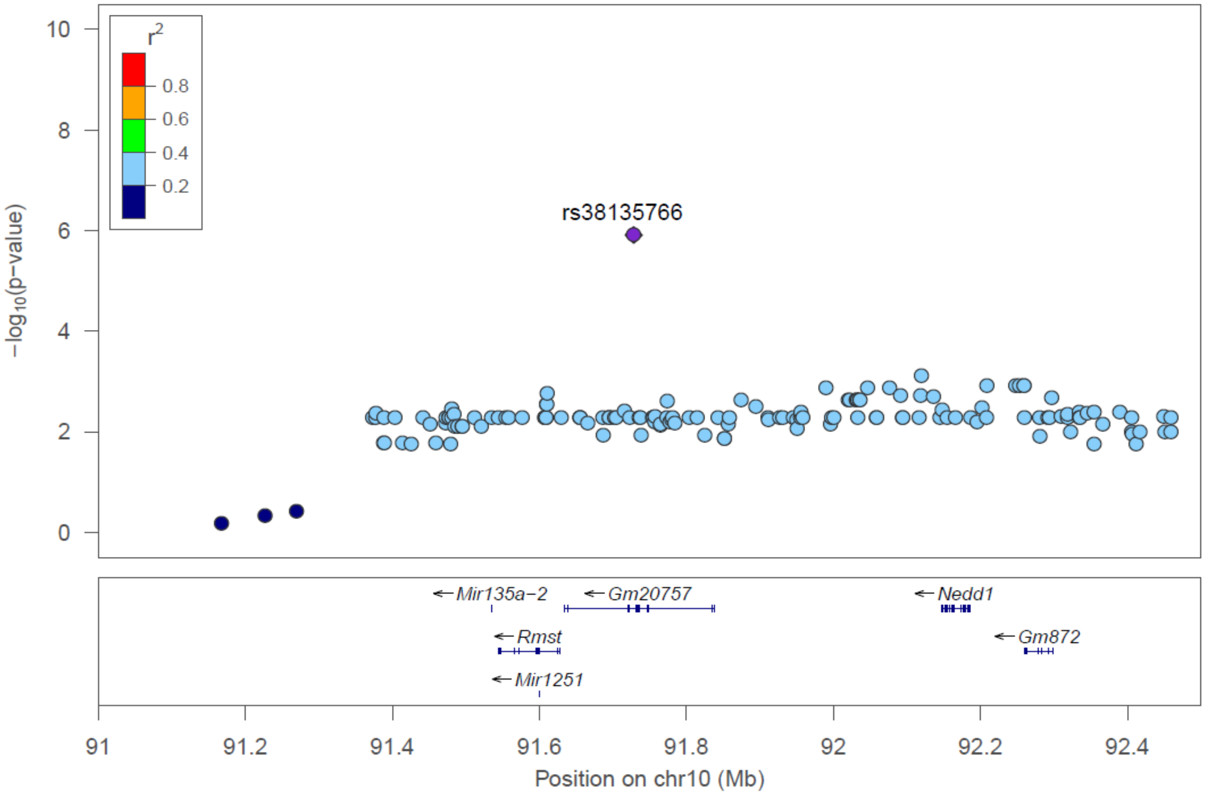

8 kHz – Chromosome 19

D

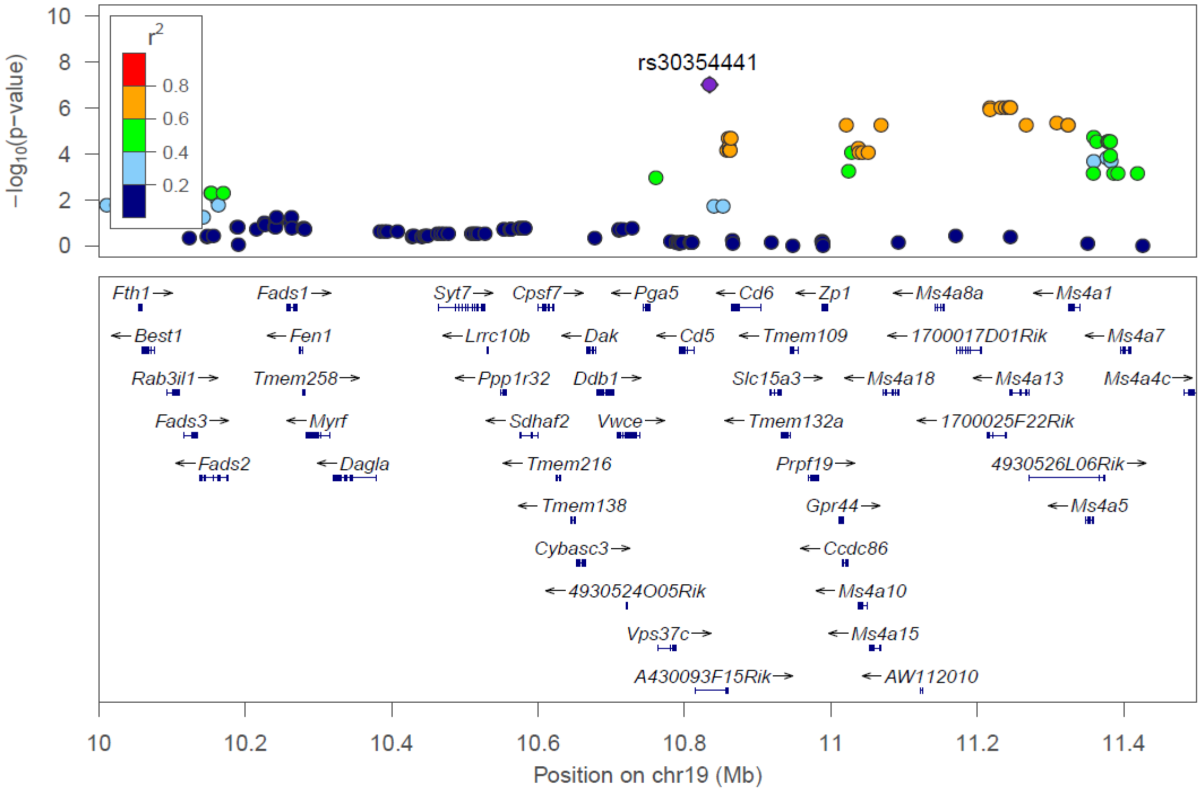

Figure S2

12 kHz – Chromosome 3

E

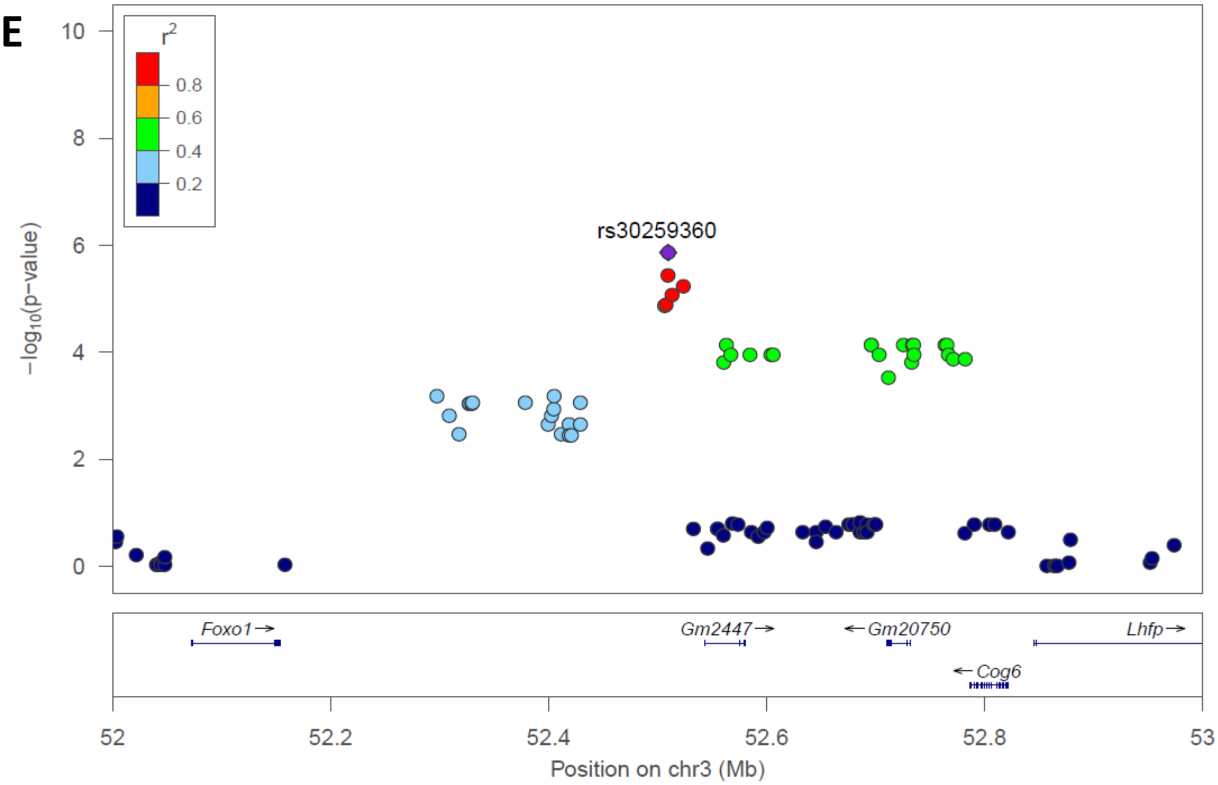

12 kHz – Chromosome 19

F

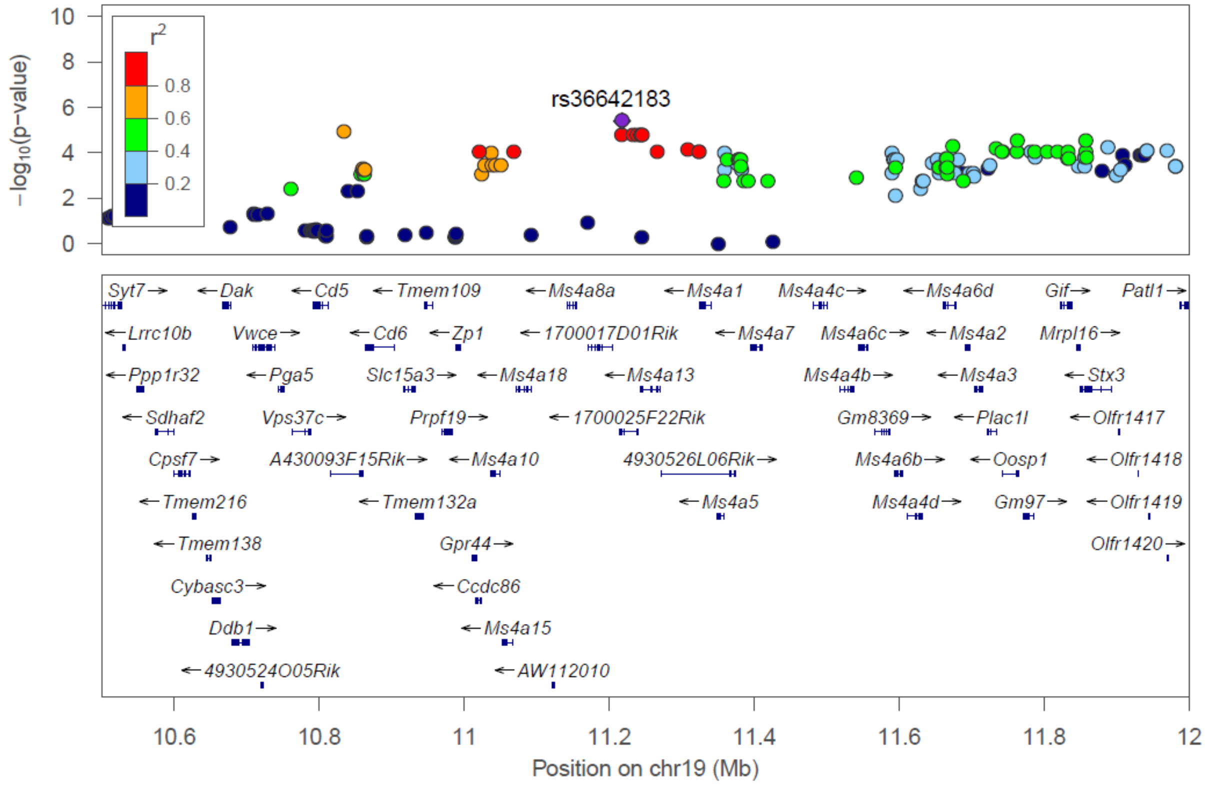

Figure S2

16 kHz – Chromosome 10

G

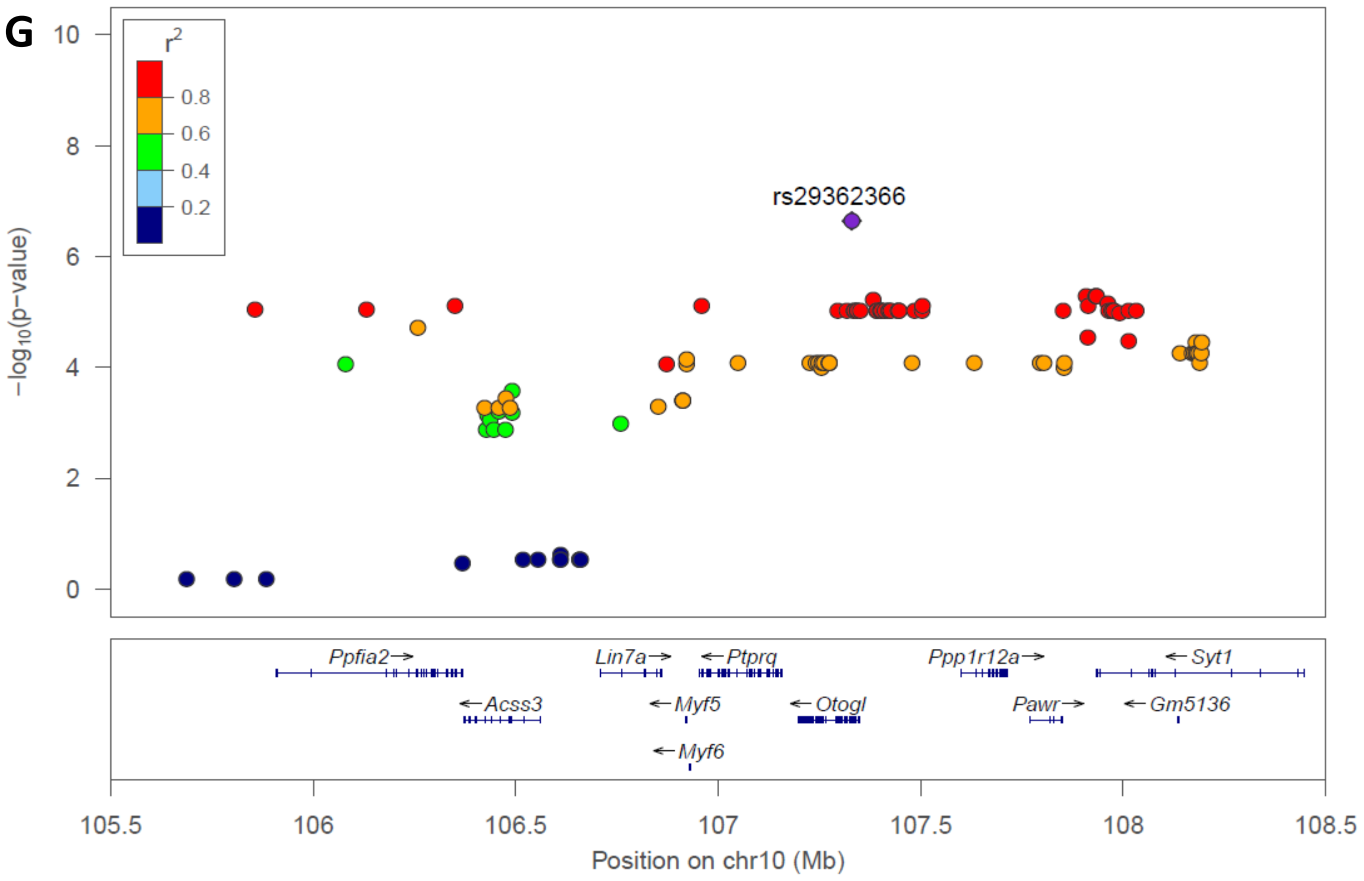

Figure S2

24 kHz – Chromosome 13

H

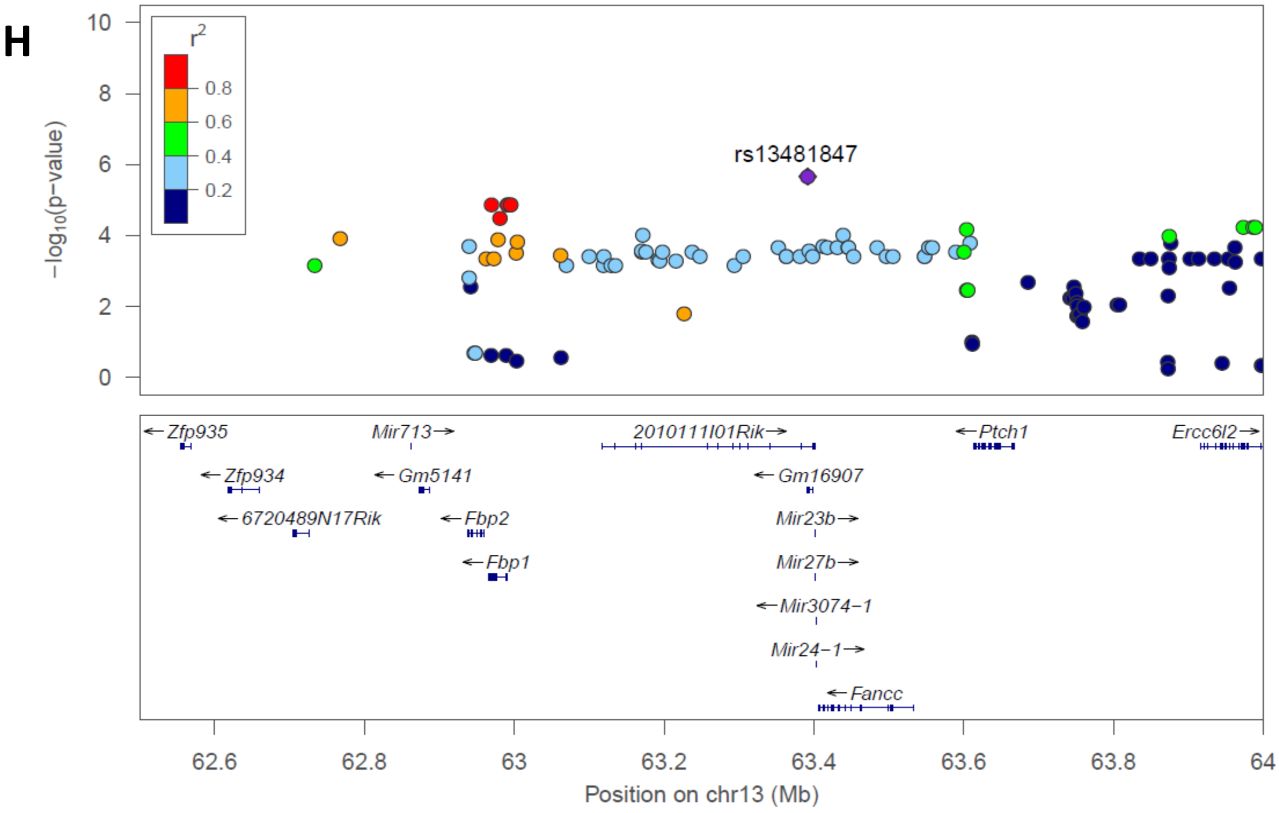

24 kHz – Chromosome 13

I

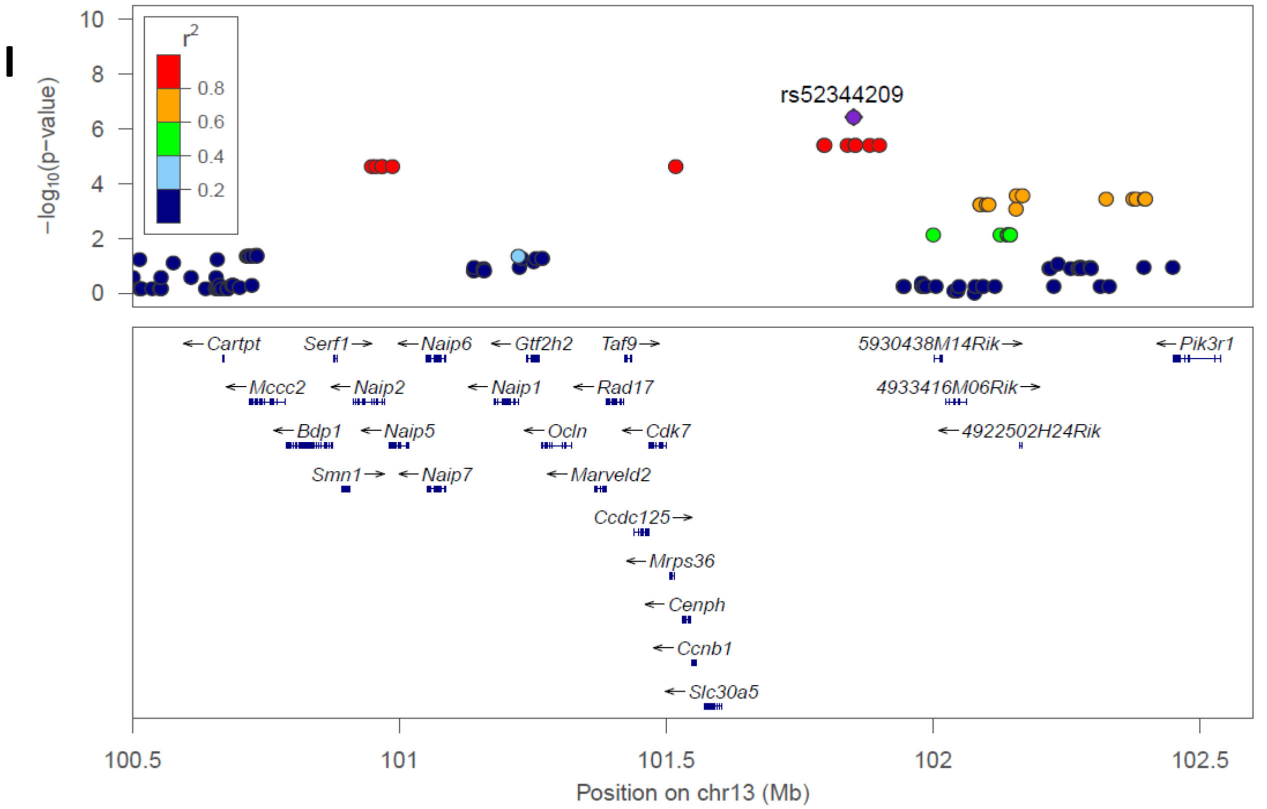

Figure S2

32 kHz – Chromosome 4

J

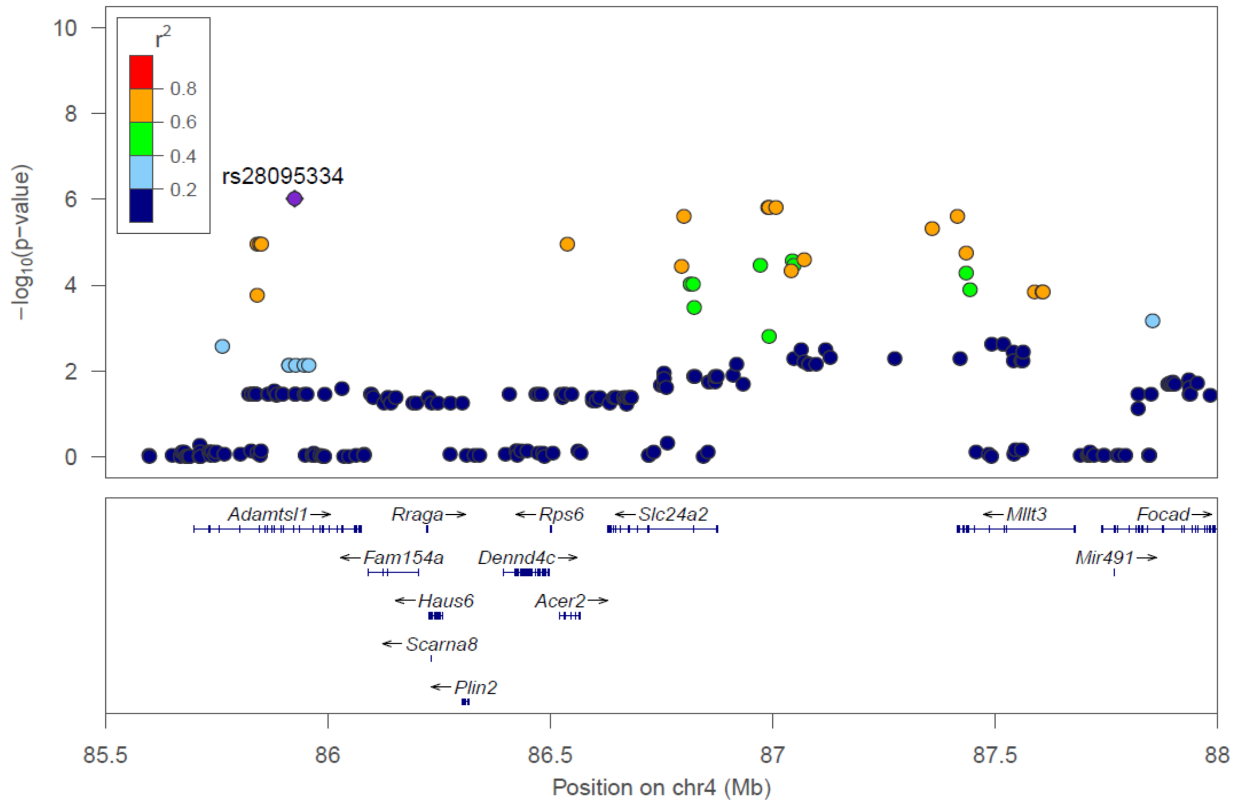

32 kHz – Chromosome 13

K

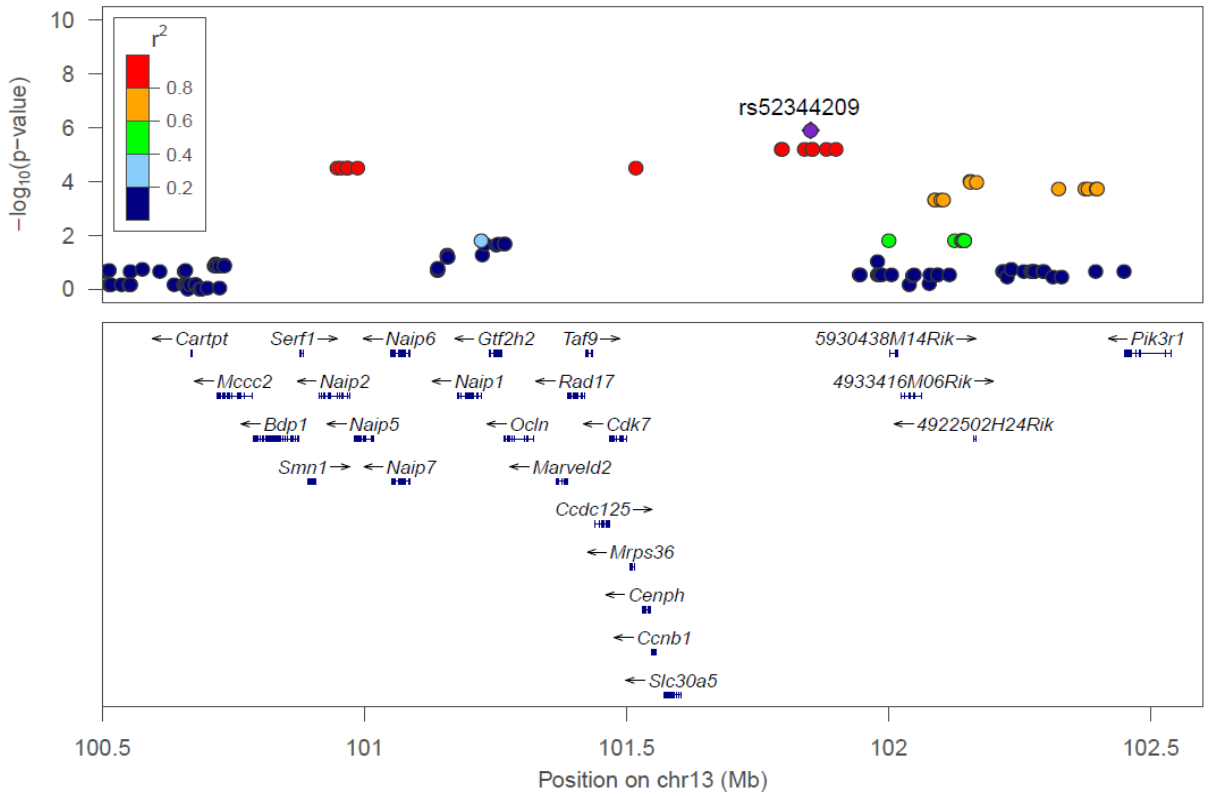

Figure S2. Regional plots of significant GWAS regions. Panels A-K present regions of significance identified by GWAS across six ABR frequencies. Boundaries are established by determining where SNPs surrounding the peak SNP are neither suggestive (at a threshold of 10^{-4}) or in moderate to high LD (r^2>=0.6) with the peak SNP.
