# Supplementary material for: The Genetic Architecture of Hearing Impairment in Mice: Evidence for Frequency-Specific Genetic Determinants
Source: G3 (Bethesda). 2015 Sep 4;5(11):2329–39. doi: 10.1534/g3.115.021592 (PMC4632053; doi:10.1534/g3.115.021592)
Supplement: Supporting Information [file supp_g3.115.021592_FigureS1.pdf]

# Figure S1

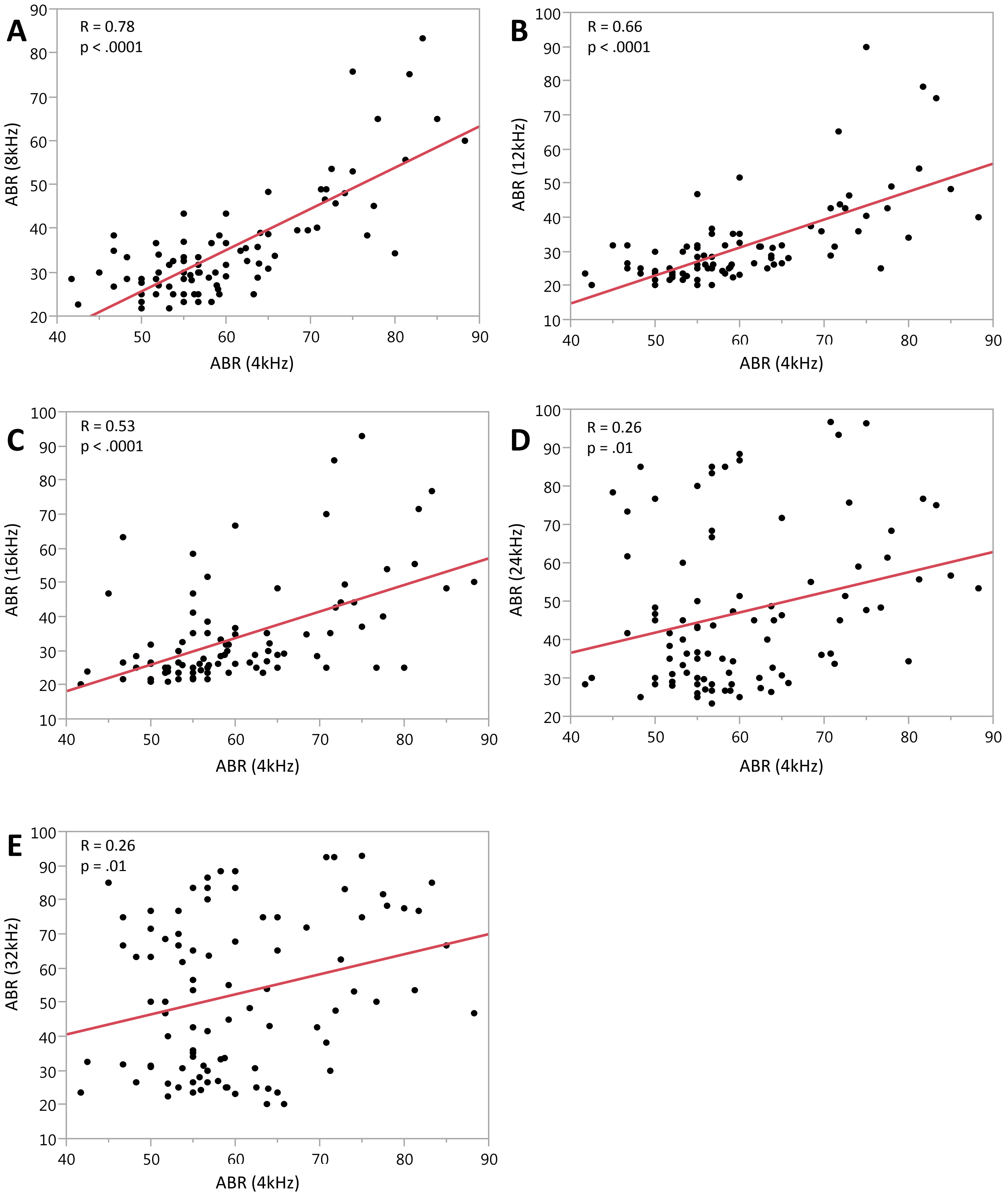
A
R = 0.78
p < .0001
ABR (8kHz)
ABR (4kHz)
B
R = 0.66
p < .0001
ABR (12kHz)
ABR (4kHz)
C
R = 0.53
p < .0001
ABR (16kHz)
ABR (4kHz)
D
R = 0.26
p = .01
ABR (24kHz)
ABR (4kHz)
E
R = 0.26
p = .01
ABR (32kHz)
ABR (4kHz)

# Figure S1

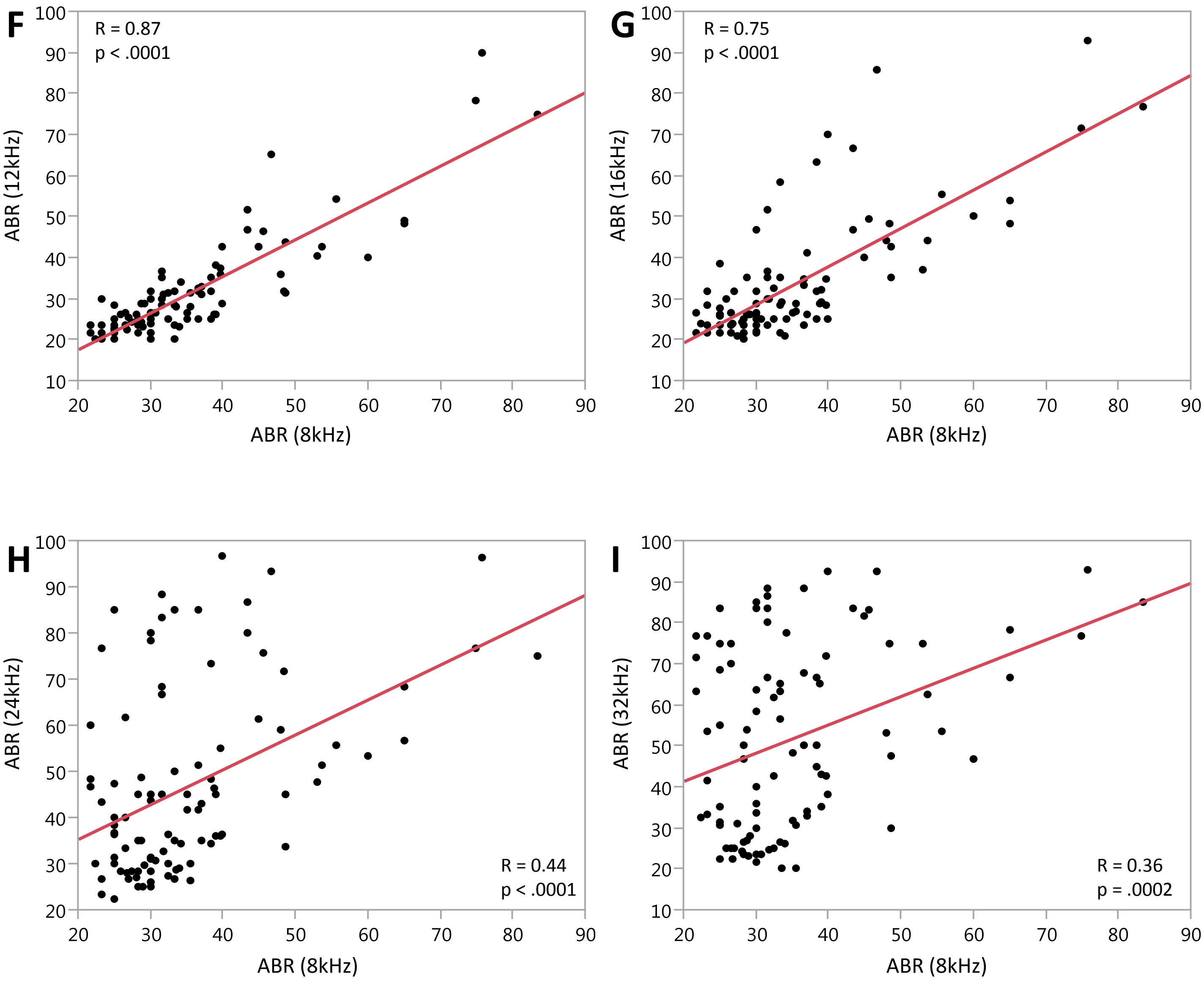
F
R = 0.87
p < .0001
ABR (12kHz)
ABR (8kHz)
G
R = 0.75
p < .0001
ABR (16kHz)
ABR (8kHz)
H
R = 0.44
p < .0001
ABR (24kHz)
ABR (8kHz)
I
R = 0.36
p = .0002
ABR (32kHz)
ABR (8kHz)

# Figure S1

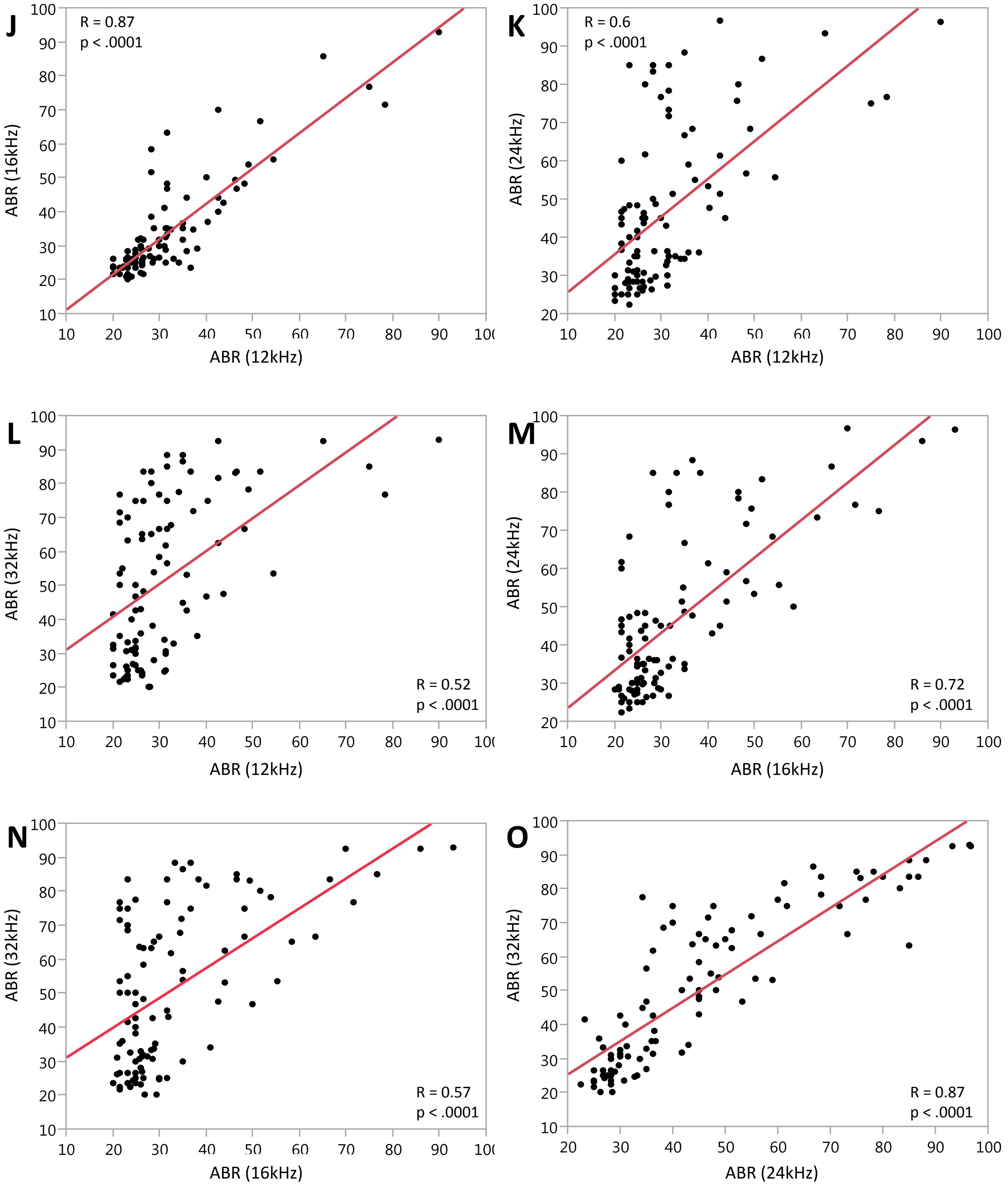
J
R = 0.87
p < .0001
ABR (16kHz)
ABR (12kHz)
K
R = 0.6
p < .0001
ABR (24kHz)
ABR (12kHz)
L
R = 0.52
p < .0001
ABR (32kHz)
ABR (12kHz)
M
R = 0.72
p < .0001
ABR (24kHz)
ABR (16kHz)
N
R = 0.57
p < .0001
ABR (32kHz)
ABR (16kHz)
O
R = 0.87
p < .0001
ABR (32kHz)
ABR (24kHz)

**Figure S1. Correlation of ABR between frequencies in the HMDP.** Panels A-O demonstrate stronger correlations between mean strain ABR at similar frequencies in the hearing spectrum. Lower (4 kHz-8 kHz), mid-range (8 kHz-12 kHz-16 kHz), and higher (24 kHz-32 kHz) frequencies correlate with each other at R >= 0.75, while comparisons between frequencies outside of their range show much weaker relationships.
