# Supplementary material for: The Genetic Architecture of Hearing Impairment in Mice: Evidence for Frequency-Specific Genetic Determinants
Source: G3 (Bethesda). 2015 Sep 4;5(11):2329–39. doi: 10.1534/g3.115.021592 (PMC4632053; doi:10.1534/g3.115.021592)
Supplement: Supporting Information [file supp_g3.115.021592_FigureS2.pdf]

Figure S2

4 kHz – Chromosome 9

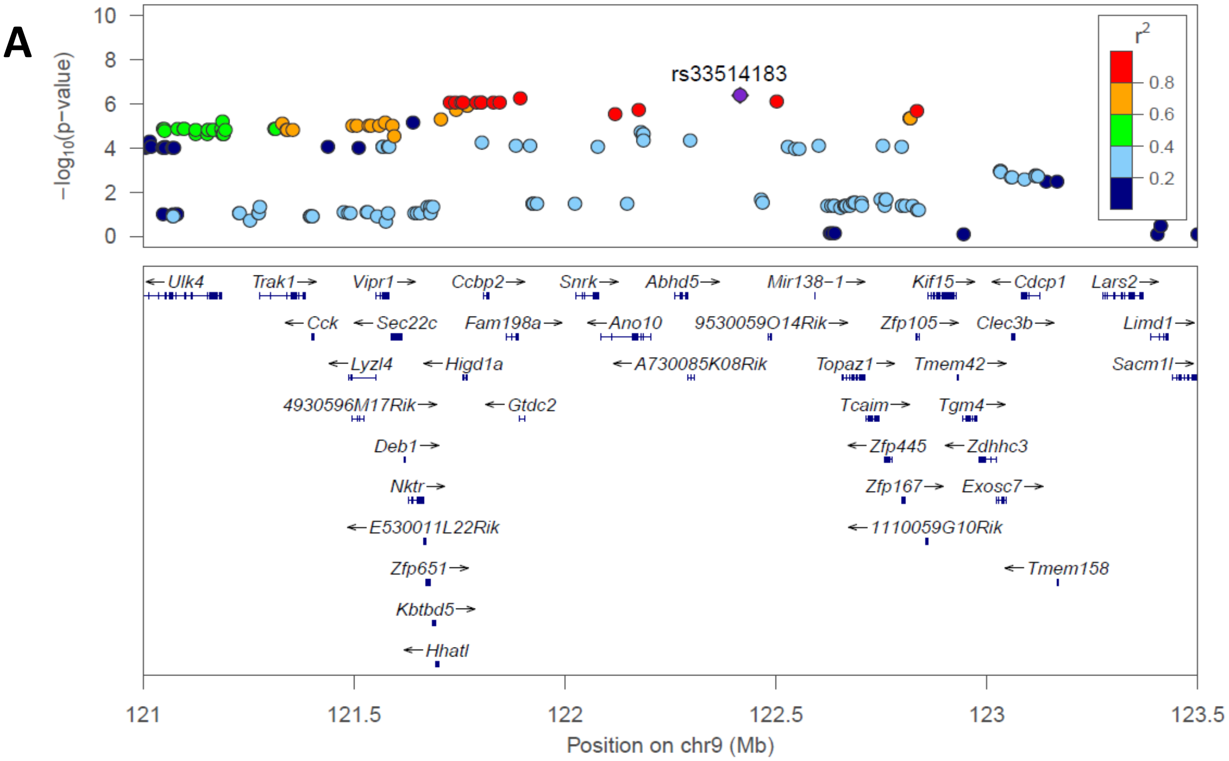

4 kHz – Chromosome 19

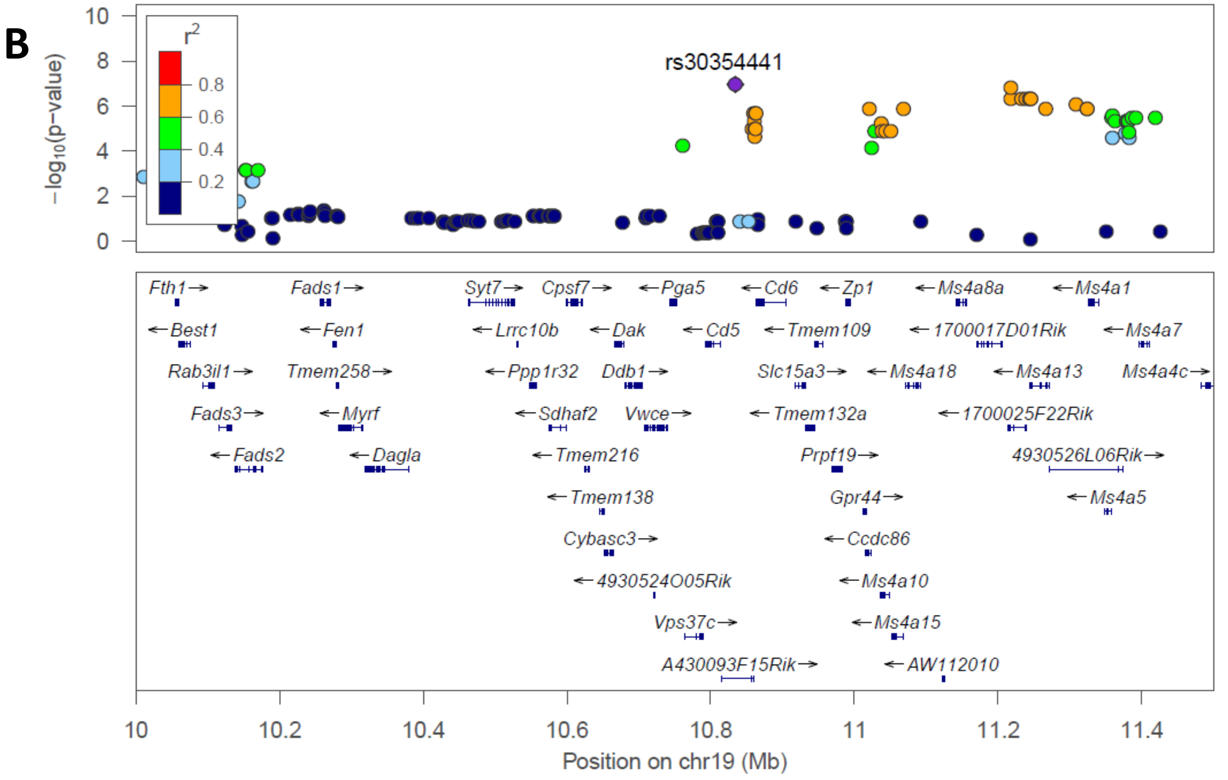

Figure S2

8 kHz – Chromosome 10

C

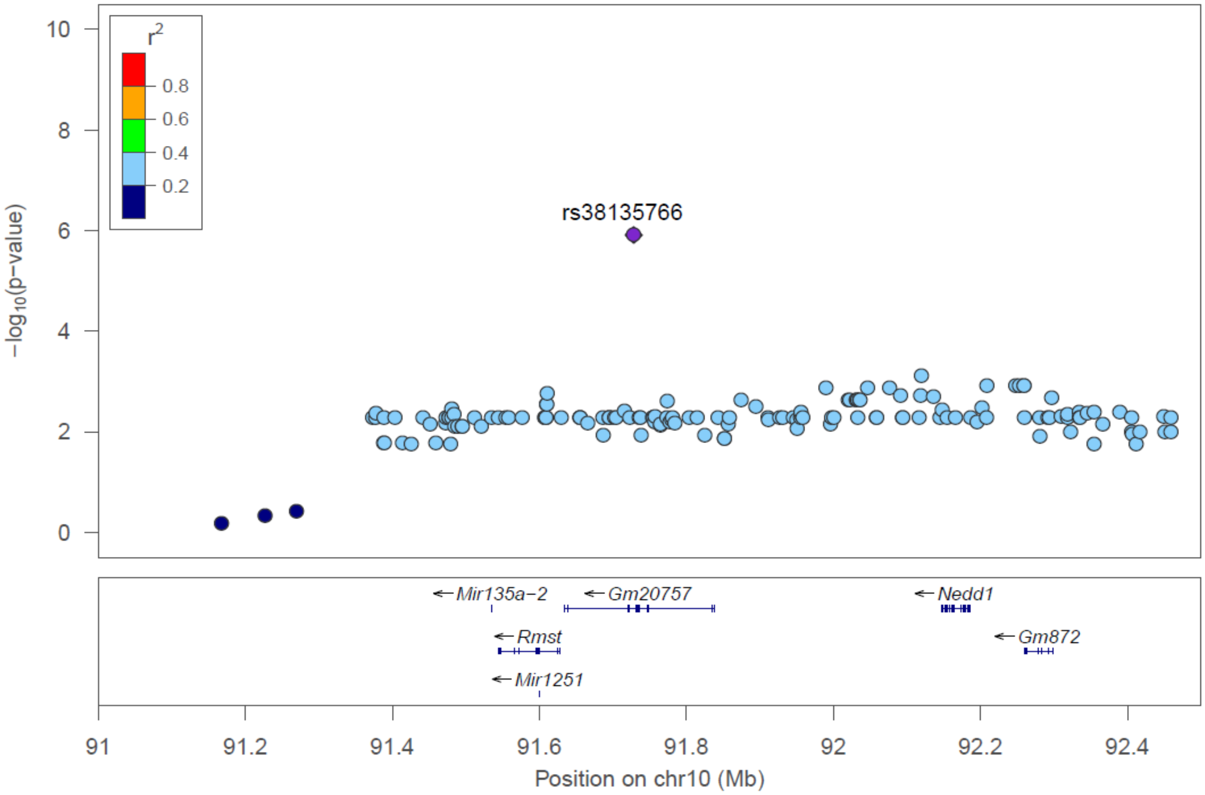

8 kHz – Chromosome 19

D

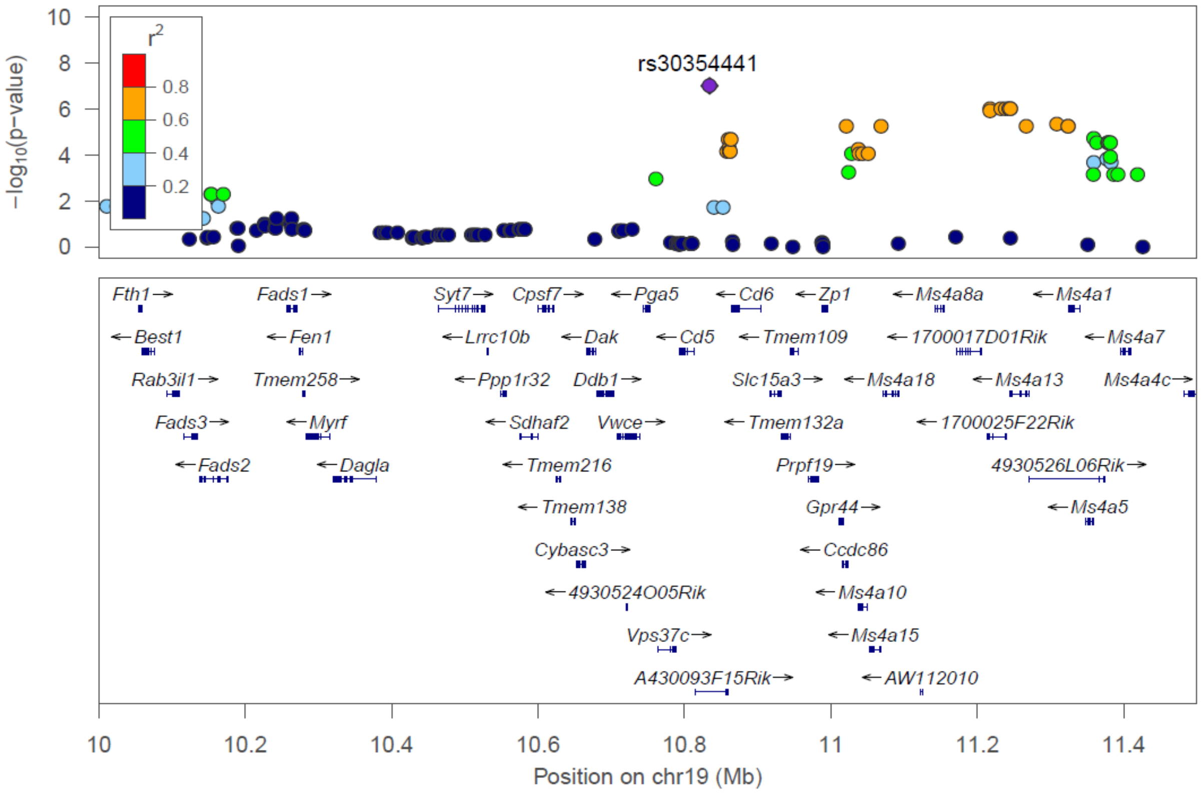

Figure S2

12 kHz – Chromosome 3

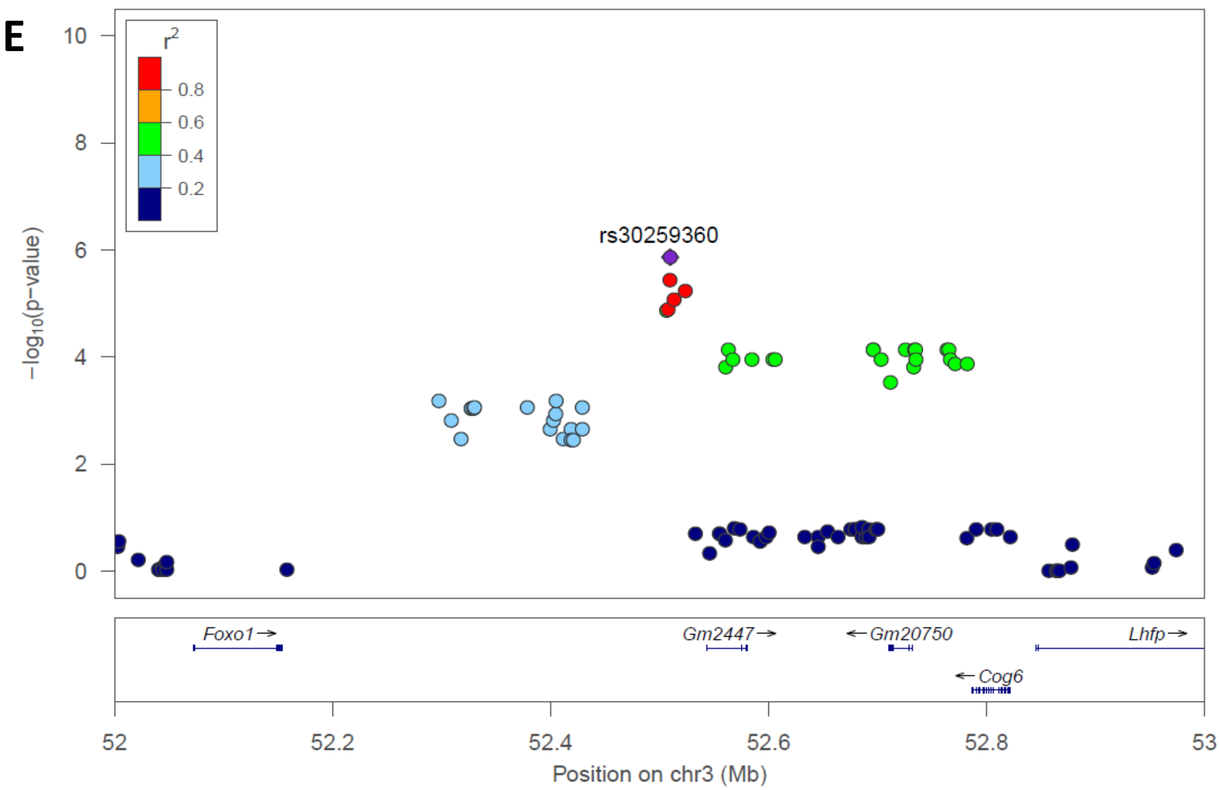

12 kHz – Chromosome 19

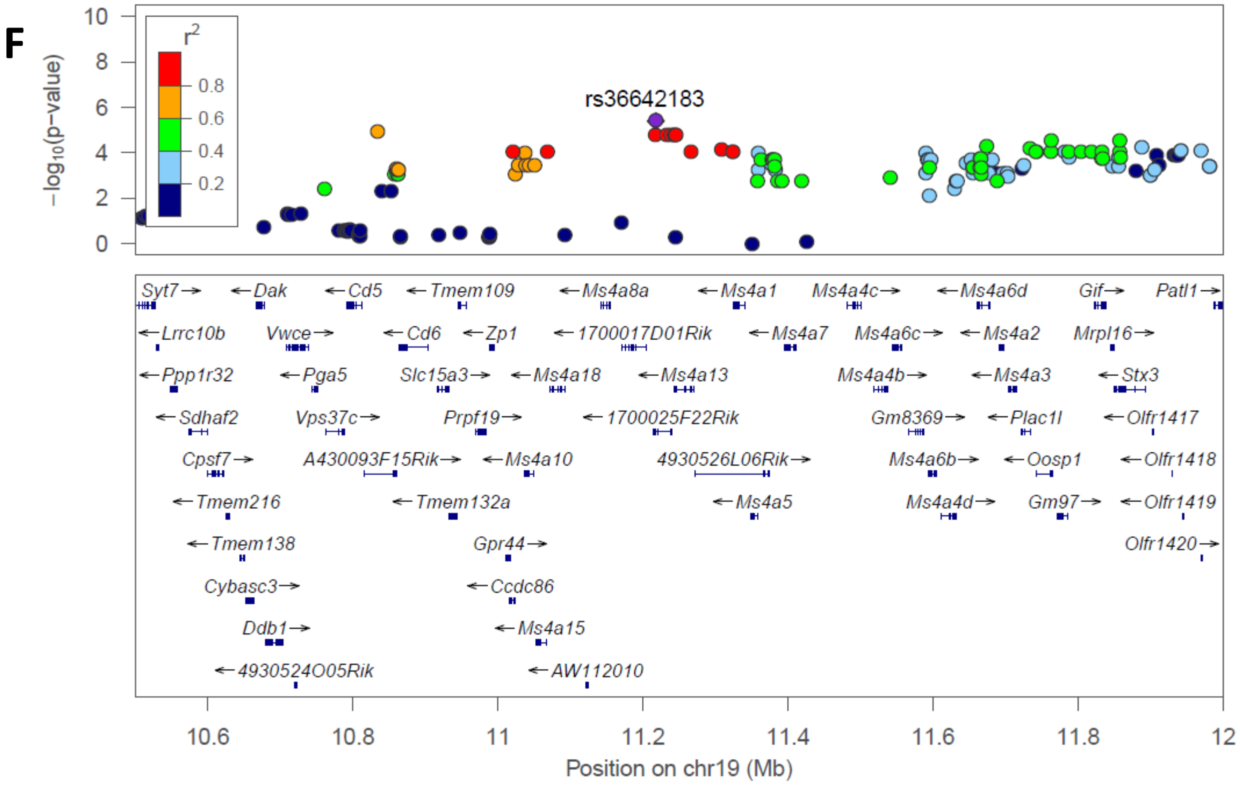

Figure S2

16 kHz – Chromosome 10

G

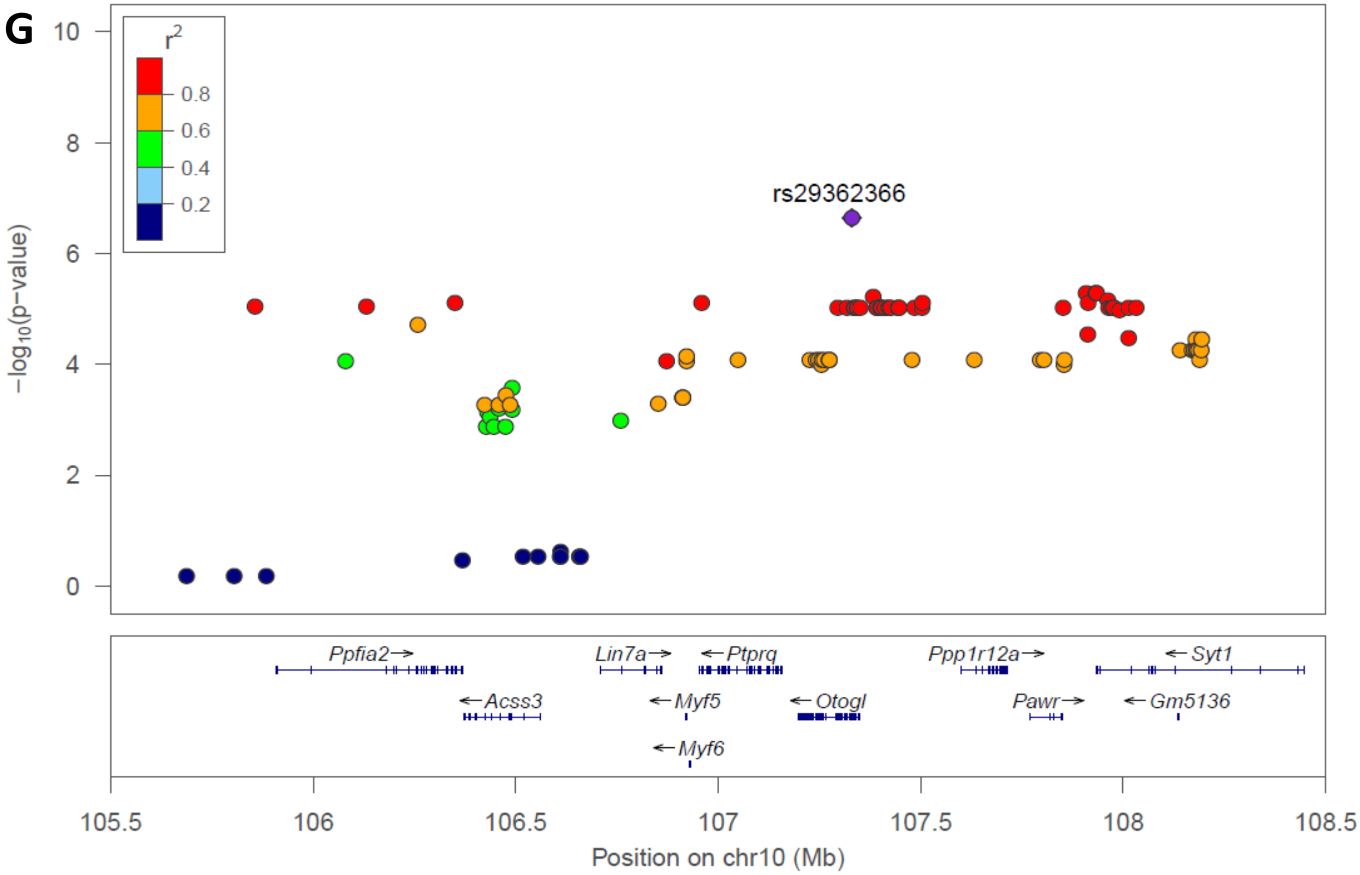

Figure S2

24 kHz – Chromosome 13

H

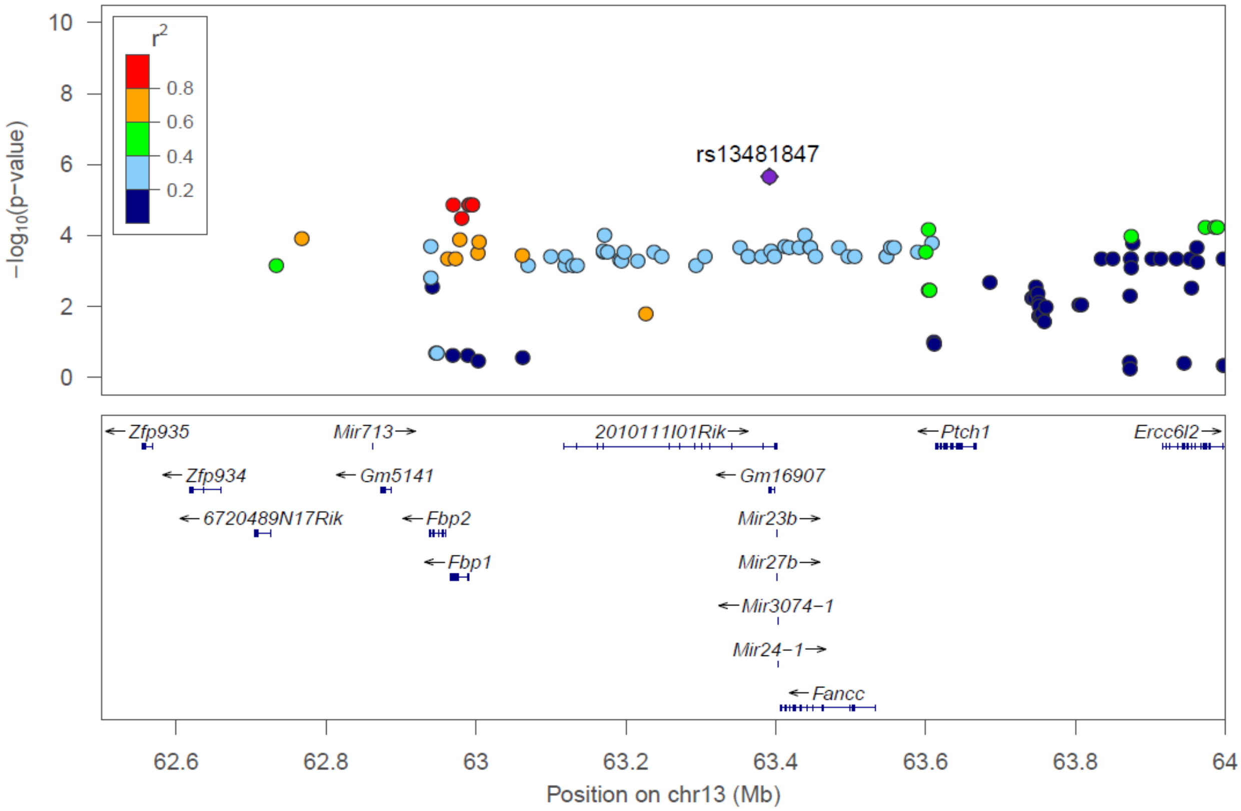

24 kHz – Chromosome 13

I

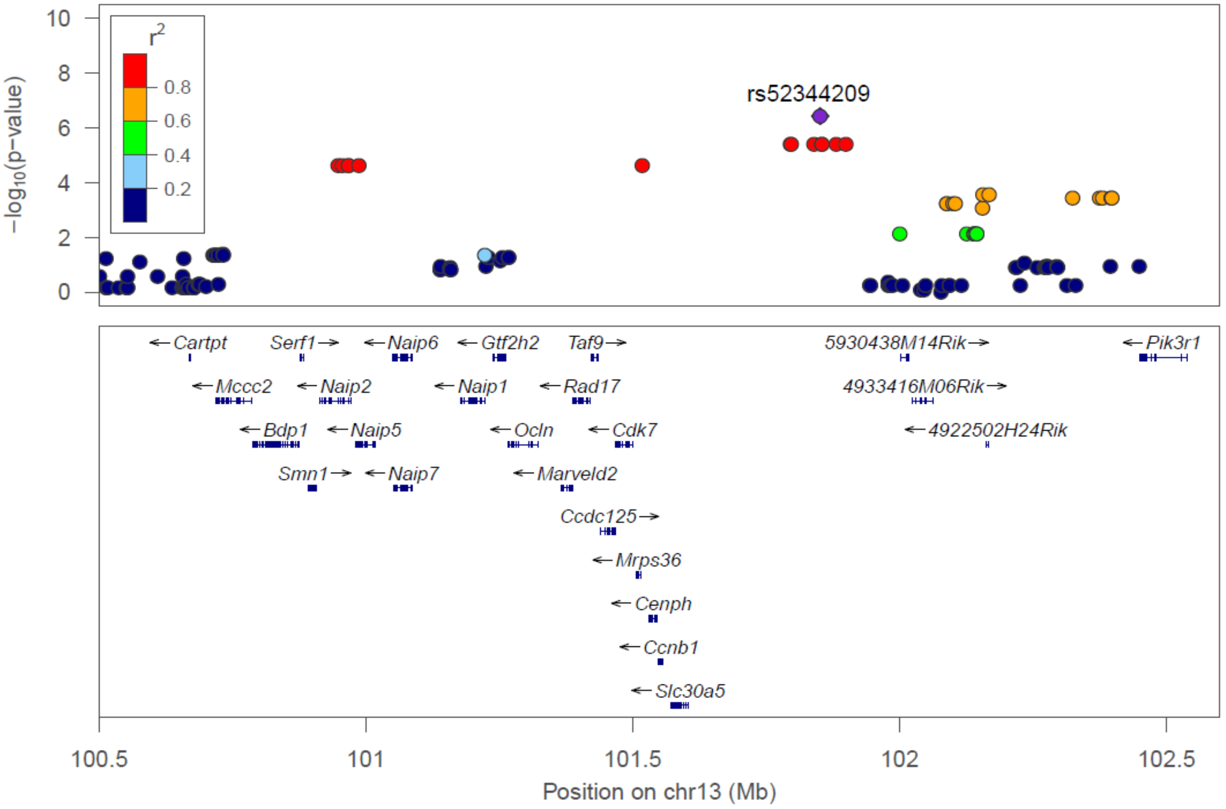

Figure S2

32 kHz – Chromosome 4

J

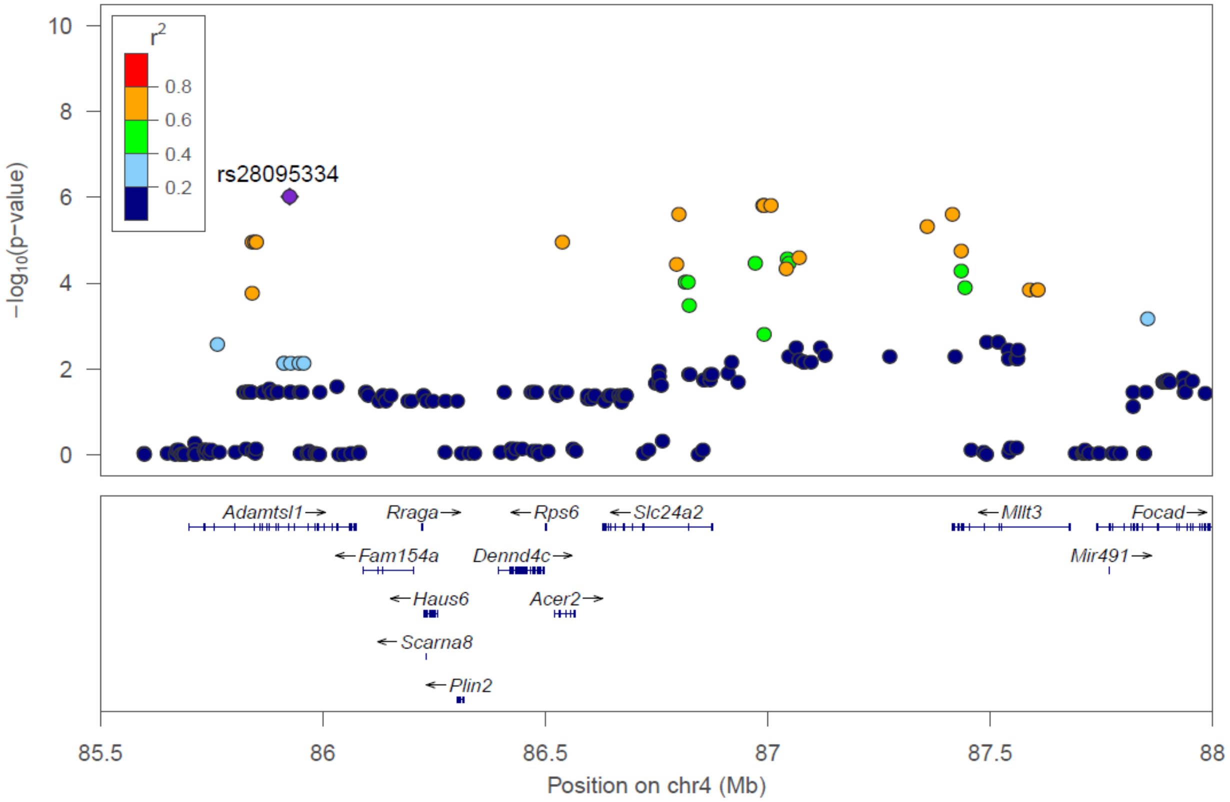

32 kHz – Chromosome 13

K

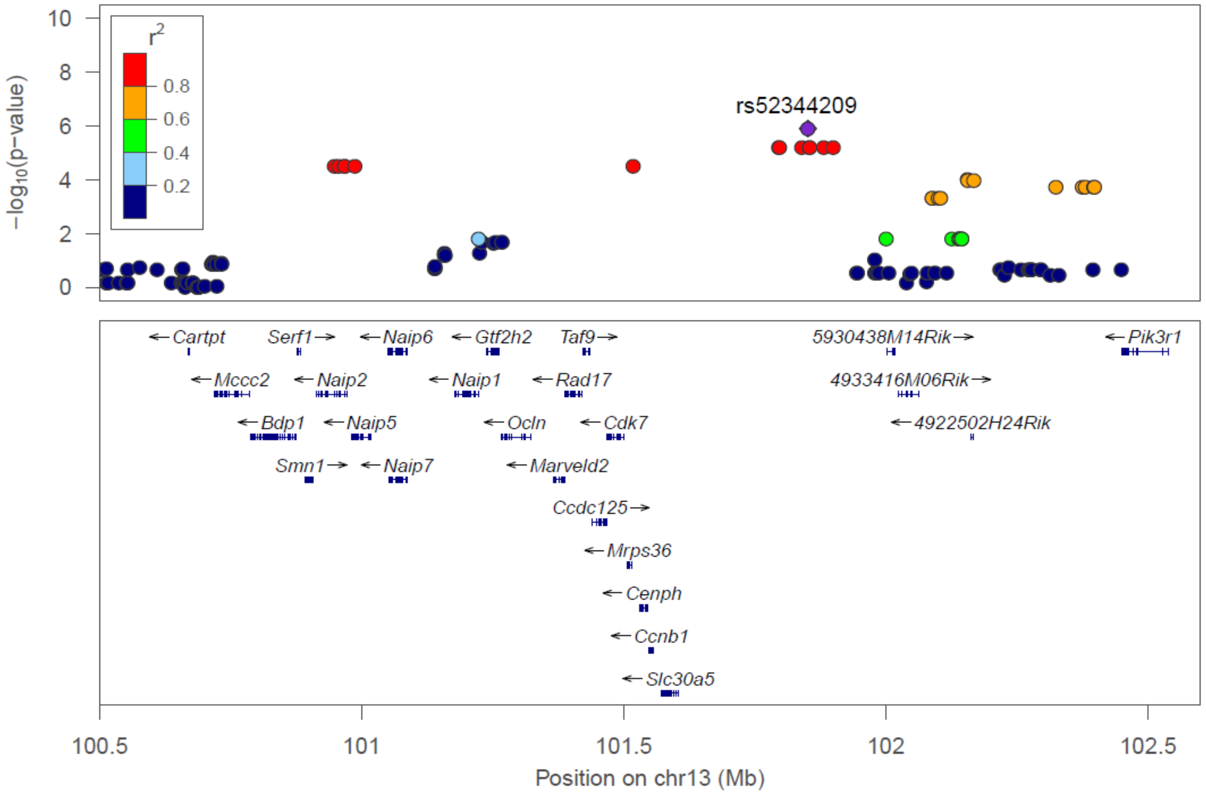

Figure S2. Regional plots of significant GWAS regions. Panels A-K present regions of significance identified by GWAS across six ABR frequencies. Boundaries are established by determining where SNPs surrounding the peak SNP are neither suggestive (at a threshold of 10^{-4}) or in moderate to high LD (r^2>=0.6) with the peak SNP.
